# The microbiota-NK cell axis in colorectal cancer: a spatial and subset-centric framework

**DOI:** 10.3389/fimmu.2026.1934819

**Published:** 2026-08-21

**Authors:** Jiexia Wen, Yunhuan Gao, Meimei Xu, Muran Li, Xuetao Dong, Min Zhao, Bin Xuan, Xiangcai Meng, Li He, Weizhi Zhang, Rui Li, Yang Tao, Fangyizhuo Zhang, Yimin Wang

**Affiliations:** 1 Department of Central Laboratory, The First Hospital of Qinhuangdao, Qinhuangdao, Hebei, China; 2 Key Laboratory of Research on Molecular Mechanism of Gastrointestinal Tumors in Qinhuangdao, The First Hospital of Qinhuangdao, Qinhuangdao, Hebei, China; 3 Joint Laboratory for Tumor Pathogenesis and Precision Medicine, The First Hospital of Qinhuangdao, State Key Laboratory of Neurology and Oncology Drug Development, Qinhuangdao, Hebei, China; 4 Department of Immunology, Nankai University School of Medicine, Nankai University, Tianjin, China; 5 Department of Gastroenterology, The First Hospital of Qinhuangdao, Qinhuangdao, Hebei, China; 6 Department of Gastroenterology, Tianjin Union Medical Center, The First Affiliated Hospital of Nankai University, Tianjin, China; 7 Department of Pathology, The First Hospital of Qinhuangdao, Qinhuangdao, Hebei, China; 8 Department of General Surgery, The First Hospital of Qinhuangdao, Qinhuangdao, Hebei, China

**Keywords:** colorectal cancer, gut microbiota, natural killer cells, spatial transcriptomics, tumor microenvironment

## Abstract

The gut microbiota and natural killer (NK) cells jointly shape colorectal cancer (CRC) progression and immunotherapy response, yet existing reviews largely adopt a linear, pathogen-centric view that overlooks NK-cell heterogeneity and spatial tumor microenvironment architecture. We propose an integrated framework centered on three progressive pillars: (i) the microbiota appears to selectively shape three well-characterized NK-cell states (activated, dysfunctional, and memory-like), with a fourth regulatory-like state that remains mechanistically uncharacterized through metabolically and genetically mediated mechanisms; (ii) emerging spatial evidence suggests beneficial commensals and activated NK cells preferentially localize to perivascular niches, whereas pathogenic bacteria and dysfunctional NK cells are enriched within the tumor parenchyma, though multicenter validation is needed; and (iii) these insights support two testable patient-stratification strategies for future clinical testing, an “enrichment” approach for tumors with pre-existing activated NK signatures, and a “remodeling” approach for Fusobacterium nucleatum (*F. nucleatum*)-dominant immunosuppressive microenvironments, particularly in refractory microsatellite-stable disease. This framework seeks to shift the field toward a subset-centric, spatially resolved, and clinically testable model of the microbiota-NK axis, designed to generate hypotheses and guide prospective cohort studies rather than replace current standards.

## 1 Introduction

Colorectal cancer (CRC) is among the most common malignancies worldwide (1–3). Patients with microsatellite-stable (MSS) tumors derive limited benefit from immune checkpoint blockade, which has intensified interest in the tumor microenvironment (TME) and tumor-associated microbiota (4–7).

The microbiota inhabiting CRC is now recognized as an active modulator of tumor progression and immune evasion (8, 9). Among immune effectors, natural killer (NK) cells constitute a first line of antitumor defense, and their functional status is closely associated with patient survival while also being profoundly shaped by local microbial cues (10–13). However, most existing reviews adopt a linear, pathogen-centric perspective that does not adequately account for NK-cell heterogeneity or the spatial organization of the tumor microenvironment (14).

This review seeks to establish an integrated, hypothesis-driven framework built on three main principles. First, it emphasizes NK-cell heterogeneity. Single-cell sequencing studies suggest that CRC tissues harbor at least three well-characterized NK-cell subsets (activated, dysfunctional, and memory-like), with a fourth regulatory-like state that remains mechanistically uncharacterized and lacks validated microbial drivers (15–18). More broadly, pan-cancer and high-dimensional single-cell studies also show that NK cells display marked transcriptional and developmental diversity across tumors, supporting the biological plausibility of functionally distinct CRC NK states (15, 19). Available evidence indicates that each subset may be linked to specific microbial signals (20–22). Second, it introduces spatial resolution as a critical dimension. Preliminary observations from small-scale spatial transcriptomics studies, most of which are single-center and include no more than 50 cases, suggest that microbial effects on NK cells depend not only on microbial identity but also on spatial localization within the tumor microenvironment (23–25). These findings should be interpreted as exploratory rather than as established biomarker thresholds (14). Third, this review moves beyond description toward a hypothesis-driven translational framework. By integrating current mechanistic and spatial insights, it proposes two precision strategies amenable to future clinical testing: an “enrichment strategy” for immunologically hot patients and a “remodeling strategy” for patients with Fusobacterium nucleatum (*F. nucleatum)*–dominant immunosuppressive microenvironments (12, 26).

Through this conceptual shift, the review aims to provide a new research roadmap for targeting the microbiota-NK cell axis in CRC, particularly in immunotherapy-resistant MSS disease (6, 27).

## 2 Four NK cell subsets: activated, dysfunctional, memory‑like, and regulatory

Before exploring how microbes modulate NK cell activity, it is important to recognize that NK cells constitute a functionally heterogeneous population. The conventional classification based on CD56 and CD16 surface expression, dividing cells into CD56^bright^ CD16^−^ immunoregulatory and CD56^dim^ CD16^+^ cytotoxic subsets, offers a foundational framework (17). However, this binary system does not adequately capture the full spectrum of functional diversity observed within the tumor microenvironment. Indeed, single-cell sequencing analyses of CRC tissues consistently reveal at least three well-characterized NK-cell subsets (activated, dysfunctional, and memory-like), with a fourth regulatory-like state that remains mechanistically uncharacterized and lacks validated microbial drivers (15, 28).

### 2.1 Activated NK cells

Activated NK cells are characterized by high expression of granzyme B (GZMB) and interferon‐γ (IFN‐γ), alongside activating receptors such as NKG2D and DNAM‑1, whereas inhibitory checkpoints including programmed cell death protein 1 (PD‑1) and T cell immunoreceptor with Ig and ITIM domains (TIGIT) remain at low levels (29, 30). These cells exhibit potent cytotoxic activity and are widely regarded as the primary effector population mediating NK cell‑dependent antitumor immunity (31). They are predominantly localized within the tumor stroma and perivascular regions (24), and their distribution and function are closely associated with beneficial gut microbes, including *Bacteroides uniformis* (*B. uniformis*) and *Lactobacillus rhamnosus* (*L. rhamnosus*), which activate NK cells through microbial products such as zwitterionic polysaccharides (ZPS) and short‑chain fatty acids (SCFAs) (26, 32). Functionally, activated NK cells exert direct antitumor effects via the secretion of GZMB, perforin, and IFN‑γ (33, 34), and additionally promote adaptive immune responses by activating dendritic cells (DCs) (35). Collectively, these features establish activated NK cells as the core subset driving antitumor immunity.

### 2.2 Dysfunctional NK cells

Dysfunctional NK cells display a phenotype largely opposite to that of their activated counterparts, characterized by elevated expression of PD‑1 and TIGIT, reduced levels of GZMB and perforin, and a propensity to secrete immunosuppressive cytokines such as interleukin‑10 (IL‑10) and transforming growth factor‑β (TGF‑β) (36, 37). Importantly, current understanding of the functional attributes of this subset in CRC is predominantly derived from single‑cell transcriptomic profiling and correlative indirect evidence (38); consequently, whether these cells actively suppress other immune populations remains to be directly validated through lineage‑tracing and functional intervention studies. Nevertheless, these dysfunctional cells appear not only to exhibit impaired tumoricidal capacity but may also, under specific microenvironmental cues, inhibit other immune effector cells (39). They are primarily enriched within the tumor parenchyma and show the strongest association with *F. nucleatum*; notably, TIGIT expression correlates positively with *F. nucleatum* abundance (12, 40).

### 2.3 Memory‑like NK cells

Memory-like NK cells are distinguished by the expression of CD45RO and CD69, and are characterized by augmented recall responses upon antigen re‑encounter (41, 42). Their maintenance appears to be supported by signals derived from *Akkermansia muciniphila* (*A. muciniphila*) through nicotinamide metabolic pathways (43, 44). Phenotypically, these cells exhibit high surface levels of the chemokine receptors CXCR3 and CCR5, alongside low expression of the adaptor protein FcϵRIγ, the inhibitory receptors NKG2A and Siglec‑7, and the glucose transporter GLUT1 (42, 45). Functionally, memory‑like NK cells demonstrate potentiated reactivity upon re‑exposure to tumor antigens, thereby contributing to the surveillance of tumor recurrence and the establishment of long‑term immune protection (41).

### 2.4 Regulatory NK cells

A fourth NK cell state, characterized by IL-10 and TGF-β production and reported to suppress activated NK and CD8^+^ T cells, has been termed “NK regulatory cells” in the literature (46). In humans, the CD56^bright^ CD16^−^ NK cell subset produces abundant cytokines upon stimulation and has been functionally analogized to CD4^+^ T helper cells, whereas the CD56^dim^CD16^+^ subset is primarily cytotoxic (46). NK cells have also been shown to limit dendritic cell-mediated T cell priming via the TRAIL pathway (47).

However, several aspects of this population remain unresolved in the context of CRC. Standardized markers for defining regulatory NK cells are lacking, and it is unclear whether they represent a stable, terminally differentiated subset or a transient state induced by specific cytokine milieus (46, 47). With respect to microbial regulation, *Bacteroides fragilis* (*B. fragilis*), the organism most frequently discussed in this context, produces Bacteroides fragilis-derived α-galactosylceramides (BfaGCs) that activate invariant natural killer T cells (iNKT) cells via CD1d (48), polysaccharide A that induces Treg differentiation through the IFNAR1–DC axis (49), and outer membrane vesicles that promote Treg-mediated tolerance via ATG16L1/NOD2-dependent autophagy (50). Short-chain fatty acids, the only microbial metabolites with direct evidence of modulating NK cell IL-10 secretion, have been shown to reduce rather than induce IL-10 production in NK-92 cells (51).

**Figure 1:**
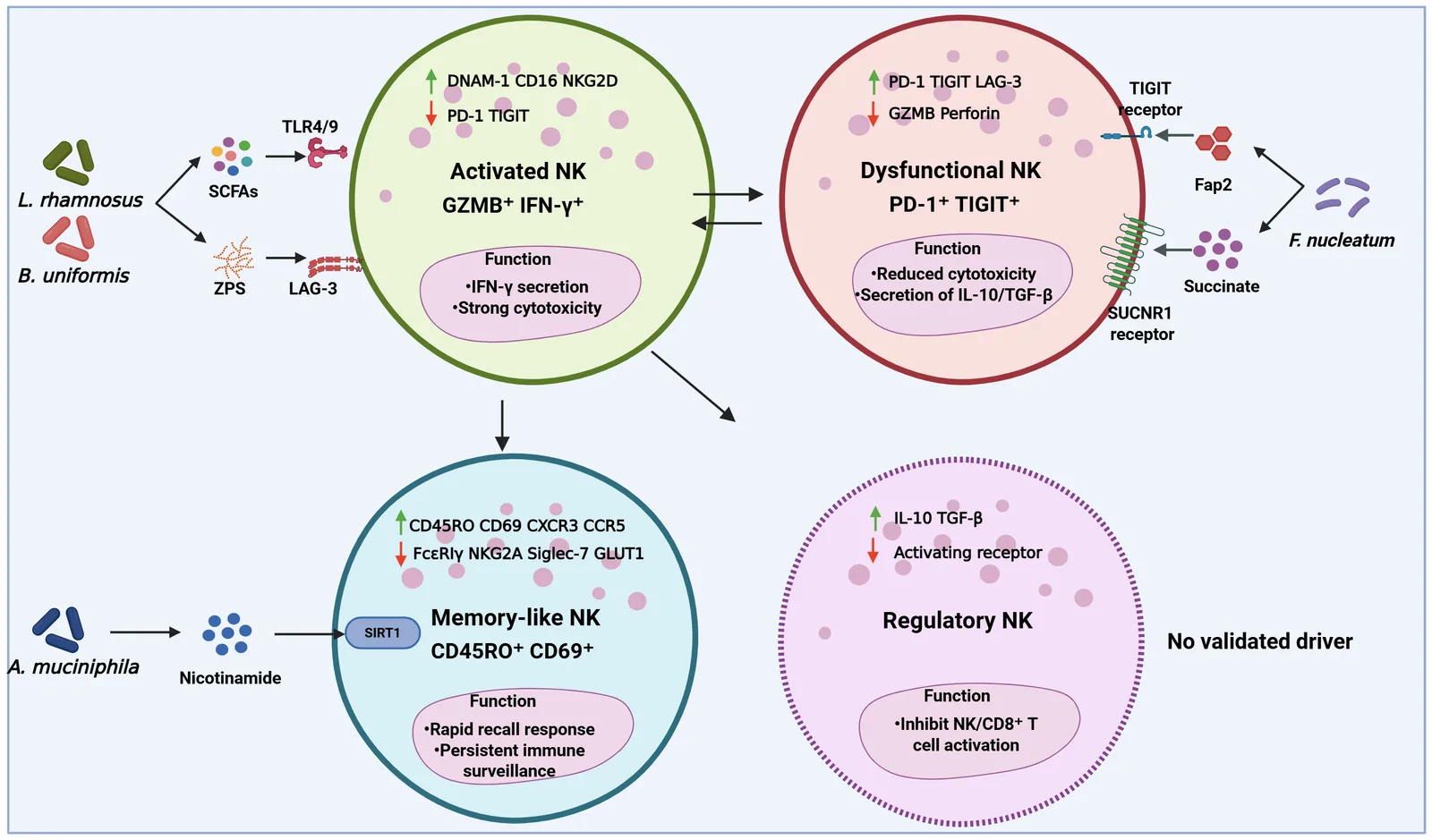
Three core NK cell subsets in colorectal cancer and their microbial drivers. Single-cell sequencing has revealed three well-characterized NK cell subsets in colorectal cancer tissues, each with unique phenotypic markers, functional characteristics, and microbial associations. Activated NK cells (GZMB^+^IFN-γ^+^, green) express high levels of activating receptors (NKG2D, DNAM-1, CD16) and are primarily driven by *B. uniformis* (via ZPS) and short-chain fatty acids (SCFAs). They exhibit strong cytotoxicity and IFN-γ secretion. Dysfunctional NK cells (PD-1^+^TIGIT^+^, red) are characterized by upregulated inhibitory receptors and reduced cytotoxic capacity, promoted by *F. nucleatum* (via Fap2 and succinate). Memory-like NK cells (CD45RO^+^CD69^+^, blue) are associated with *A. muciniphila*-derived nicotinamide and provide long-term immune surveillance. A fourth regulatory NK cells (purple, dashed box) has been described but lacks validated microbial drivers and standardized phenotypic markers, and is therefore not integrated into the core framework. Arrows indicate potential subset transitions: activated NK cells can differentiate into dysfunctional, memory-like, or regulatory subsets under specific microbial and metabolic cues.

Collectively, the available evidence identifies a phenotypically described IL-10^+^ NK cell population whose microbial regulatory pathways, where investigated, primarily engage iNKT and Treg cells rather than NK cells directly. Whether this population represents a distinct, microbe-driven NK subset in CRC therefore remains an open question (**Figure 1**).

The key characteristics of these four NK cell subsets are summarized in **Table 1**.

**Table 1:** Summary of characteristics of the four NK cell subsets.

Subset	Phenotypic markers	Functional features	Spatial distribution	Associated microbes	Ref.
Activated	GZMB^+^IFN γ^+^, NKG2D ^high^, PD-1^low^, TIGIT ^low^	High cytotoxicity, perforin/granzyme B secretion, Antibody dependent cell mediated cytotoxicity (ADCC)	Tumor stroma, perivascular niches	*B. uniformis* *L. rhamnosus*	(26, 29–34)
Dysfunctional	PD-1^+^TIGIT^+^, TIM-3^+^, GZMB ^low^, Perforin ^low^	Loss of cytotoxicity, IL-10/TGF-β secretion, metabolic reprogramming	Tumor parenchyma (adjacent to cancer cells)	*F. nucleatum*	(36–39)
Memory-like	CD45RO^+^CD69^+^, CXCR3^+^, CCR5^+^, GLUT1 ^low^	Enhanced recall responses; nicotinamide Sirtuin 1 (SIRT1) axis supports long term survival; initial differentiation signals unclear	Lymphoid like structures	*A. muciniphila* (correlative)	(41–45)
Regulatory	IL-10^+^TGF-β^+^ (definition varies across studies)	Suppresses activated NK and CD8^+^ T cells; phenotypic stability unclear	Not established	*None directly validated*	(46, 47, 50, 51)

## 3 Microbiota‑driven inhibitory axes: mechanisms of dysfunctional NK cell induction

Among the many microbial signals affecting NK cells, two inhibitory axes are relatively well characterized, both promoting the conversion of NK cells toward a dysfunctional phenotype.

### 3.1 The Fap2–TIGIT axis

The most extensively characterized inhibitory pathway in microbe-NK cell interactions is the Fap2-TIGIT axis, centered on *F. nucleatum*, an anaerobic bacterium consistently enriched in CRC tissues (52, 53). Mechanistic studies, based on animal models and *in vitro* experiments, have demonstrated that the Fap2 protein expressed on the surface of *F. nucleatum* can directly bind to the TIGIT receptor on NK cells, an inhibitory receptor belonging to the immunoglobulin superfamily. This engagement recruits SHP‑1 and SHP‑2 phosphatases, suppresses the activation of ZAP70/Syk family kinases, and consequently blocks downstream PI3K/AKT and MAPK signaling pathways, ultimately leading to reduced expression of granzyme B and perforin (54, 55). Concurrently, Fap2–TIGIT interaction induces NK cells to secrete IL‑10 and TGF‑β, further reinforcing the immunosuppressive tumor microenvironment (55). In animal models, this axis has been shown to drive the conversion of activated NK cells toward a PD‑1^+^TIGIT^+^ dysfunctional phenotype (56, 57). At the correlative level in humans, CRC patients with high *F. nucleatum* abundance exhibit significantly elevated proportions of TIGIT^+^ dysfunctional NK cells within tumor tissues, as well as shortened progression‑free survival and overall survival, an association particularly prominent in the MSS patient subgroup (57–59). Furthermore, *F. nucleatum* can synergize with tumor-associated fungi such as *Candida albicans* (*C. albicans*); although the precise molecular mechanism remains to be fully elucidated, their co-occurrence in the tumor microenvironment is associated with enhanced immunosuppression and amplification of NK cell inhibition (60).

However, it must be emphasized that, although the Fap2-TIGIT interaction has been robustly validated in mouse models and *in vitro* coculture systems (12), direct causal evidence in CRC remains indirect. All human data available to date are correlative (40, 58), and the possibility of reverse causality, whereby a dysfunctional NK microenvironment itself promotes *F. nucleatum* overgrowth, cannot be excluded. Moreover, given that TIGIT is also expressed on regulatory T cells and CD8^+^ T cells (56), the relative contributions of NK‑specific versus T‑cell‑mediated effects to the overall immunosuppressive phenotype remain to be disentangled through cell‑type‑specific gene knockout or blocking antibody experiments.

Beyond direct Fap2-TIGIT engagement, *F. nucleatum* reinforces immunosuppression through the recruitment of Myeloid-Derived Suppressor Cells (MDSCs) and M2-type Tumor-Associated Macrophages (TAMs), which together amplify NK cell dysfunction via both soluble mediators and physical barriers (see Section 6.2 for a detailed discussion of NK-myeloid antagonism under dysbiosis). These bystander mechanisms, superimposed on direct Fap2-TIGIT inhibition, constitute a multi-layered and synergistic immunosuppressive network that substantially complicates single-agent intervention strategies.

### 3.2 The succinate–SUCNR1–HIF‑1α–EZH2 axis

The second inhibitory axis is based on a metabolite rather than a surface protein, namely the succinate receptor 1 (SUCNR1)–hypoxia‑inducible factor 1 alpha (HIF‑1α)–EZH2 pathway, driven predominantly by the metabolic activity of *F. nucleatum* (61, 62). Mechanistically, *F. nucleatum* produces large amounts of succinate through its metabolism, which accumulates in tumor microenvironment and binds to SUCNR1 on NK cells (63). This binding, mediated by Gi protein‑coupled receptor signaling, inhibits cAMP accumulation while concurrently activating the PI3K/AKT pathway and inducing stabilization of HIF‑1α (61). Stabilized HIF‑1α directly binds to hypoxia response elements in the *EZH2* promoter region, promoting transcriptional upregulation of EZH2 (64). As a histone H3K27 methyltransferase, EZH2 catalyzes H3K27me3 modifications at the *cGAS* promoter, thereby suppressing cGAS–IFN‑β pathway activation, which ultimately leads to reduced IFN‑γ secretion and impaired cytotoxic activity of NK cells (65). Simultaneously, HIF‑1α promotes the expression of immune checkpoint molecules such as PD‑1 and TIGIT on NK cells, further driving the dysfunctional phenotype (66).

In addition, succinate promotes glycolysis in CRC cells, and the resulting lactate exerts context-dependent effects on NK cell function within the acidic tumor microenvironment. In most settings, lactate is recognized as a potent immunosuppressive metabolite that directly impairs NK cell metabolism, suppresses the PI3K/AKT pathway, reduces IFN-γ and granzyme B production, and promotes NK cell apoptosis, thereby favoring the expansion of dysfunctional NK subsets (67, 68). However, the net effect of lactate on NK cells is not unidirectional. Emerging evidence indicates that the functional outcome is highly dependent on local concentration, the duration of exposure, and the metabolic state of the target NK cells. At lower concentrations or under specific microenvironmental conditions, lactate may serve as an alternative fuel source that supports NK cell survival and sustains basal effector functions, suggesting that the threshold between metabolic support and suppression is contextually defined (69). Furthermore, recent studies have demonstrated that lactate can drive NK cell dysfunction through histone lactylation-mediated epigenetic reprogramming, a mechanism that operates independently of pH changes, highlighting that lactate-mediated inhibition involves both pH-dependent and pH-independent pathways (70–72). Collectively, the succinate-lactate axis should be viewed not as a simple inhibitory network but as a complex, concentration- and context-dependent metabolic rheostat that fine-tunes NK cell functional states in the CRC microenvironment. Deciphering the precise conditions that determine whether lactate suppresses or sustains NK cell activity remains a critical priority for developing targeted immunometabolic interventions (73).

At the human correlative and therapeutic potential level, succinate levels in CRC tissues correlate positively with *F. nucleatum* abundance and are associated with reduced NK cell infiltration and dysfunction. This axis is attractive as a therapeutic target because it is amenable to small molecule inhibitor development; preclinical studies have shown that SUCNR1 antagonists can effectively reverse NK cell dysfunction in the presence of *F. nucleatum* (62, 74, 75).

Nevertheless, it should be noted that the core evidence for this axis currently derives mainly from murine tumor models and *in vitro* culture systems, and direct validation in prospective CRC cohorts is lacking, a key evidence gap constraining clinical translation.

## 4 Microbiota‑driven activating axes: induction and maintenance of activated and memory‑like NK cells

### 4.1 Axes driving activated NK cells

#### 4.1.1 The ZPS–LAG-3 axis (a paradox)

Among microbial‑driven NK cell activation pathways, the *B. uniformis*‑derived Zwitterionic capsular polysaccharide (ZPS)—Lymphocyte activation gene 3 (LAG-3) axis presents a unique and paradoxical mechanism. *B. uniformis*, a beneficial bacterium associated with a healthy gut microbiota and favorable cancer prognosis, produces the capsular polysaccharide ZPS, which in mouse models can bind to the LAG-3 molecule on NK cells (26, 76). In stark contrast to the well‑established inhibitory role of LAG-3 on T cells, this binding on NK cells activates an antitumor program. Specifically, it signals through the unique adaptor protein FcϵRIγ, rather than the SHP‑1/SHP‑2 phosphatases used in T cells, and subsequently activates NF‑κB and JAK/STAT3 pathways. This signaling cascade upregulates the expression of chemokine receptors CXCR3 and CCR5, enhances NK cell responsiveness to CXCL10 and CCL5 chemokines secreted by tumors, and promotes NK cell recruitment and infiltration into the tumor microenvironment (26, 77, 78). Concurrently, it upregulates GZMB and IFN‑γ expression, maintains the proportion of activated (GZMB^+^IFN‑γ^+^) NK cells, and inhibits their conversion toward the dysfunctional phenotype (26).

The mechanistic basis of this LAG-3 paradox remains incompletely understood. The opposing effects of LAG-3 on NK cell activation versus T cell suppression may arise from differential adaptor protein usage, post‑translational modifications, or distinct ligand binding affinities on the two cell types; resolving this paradox is widely regarded as one of the most important open scientific questions in the field (26, 76). At the human correlative level, CRC patients with high *B. uniformis* abundance exhibit elevated proportions of LAG-3^+^ NK cells within tumor tissues, better overall prognosis, and a positive correlation with immunotherapy response (26, 78, 79). However, it should be emphasized that direct mechanistic evidence for the ZPS-LAG-3 activation pathway currently derives exclusively from mouse models; whether an identical signaling pathway operates on human NK cells has not yet been formally validated.

In addition, ZPS indirectly enhances NK cell function through bystander effects. It promotes polarization of tumor‑associated macrophages toward the M1 phenotype, and these M1 macrophages subsequently secrete IL‑12, which further potentiates NK cell activation. Moreover, ZPS fragments can be presented by dendritic cells to promote NK cell memory formation. Collectively, these mechanisms amplify the antitumor effects of the direct activation pathway (11, 20).

#### 4.1.2 The SCFA–TLR–MAPK/NF‑κB axis

Among microbial metabolite‑mediated NK cell regulatory mechanisms, SCFAs, primarily produced by beneficial bacteria such as *L. rhamnosus* and *B. uniformis*, including acetate, propionate, and butyrate (80, 81), bind to Toll like receptor 4 (TLR4) and Toll like receptor 9 (TLR9) on NK cells and, through Myeloid differentiation primary response 88 (MyD88)‑dependent signaling, activate downstream MAPK (comprising ERK, JNK, and p38) and NF‑κB pathways, thereby promoting NK cell proliferation and activation (82, 83). Simultaneously, SCFAs upregulate the expression of chemokines CXCL10 and CXCL11 as well as their receptor CXCR3, thereby enhancing directional NK cell recruitment to tumor sites. Among these metabolites, butyrate additionally inhibits histone deacetylase (HDAC) activity, increasing histone acetylation at the granzyme B and perforin promoter regions and consequently enhancing their transcriptional expression (84, 85); propionate and acetate act synergistically to elevate intracellular cAMP levels, further reinforcing the activated state of NK cells (68).

**Figure 2:**
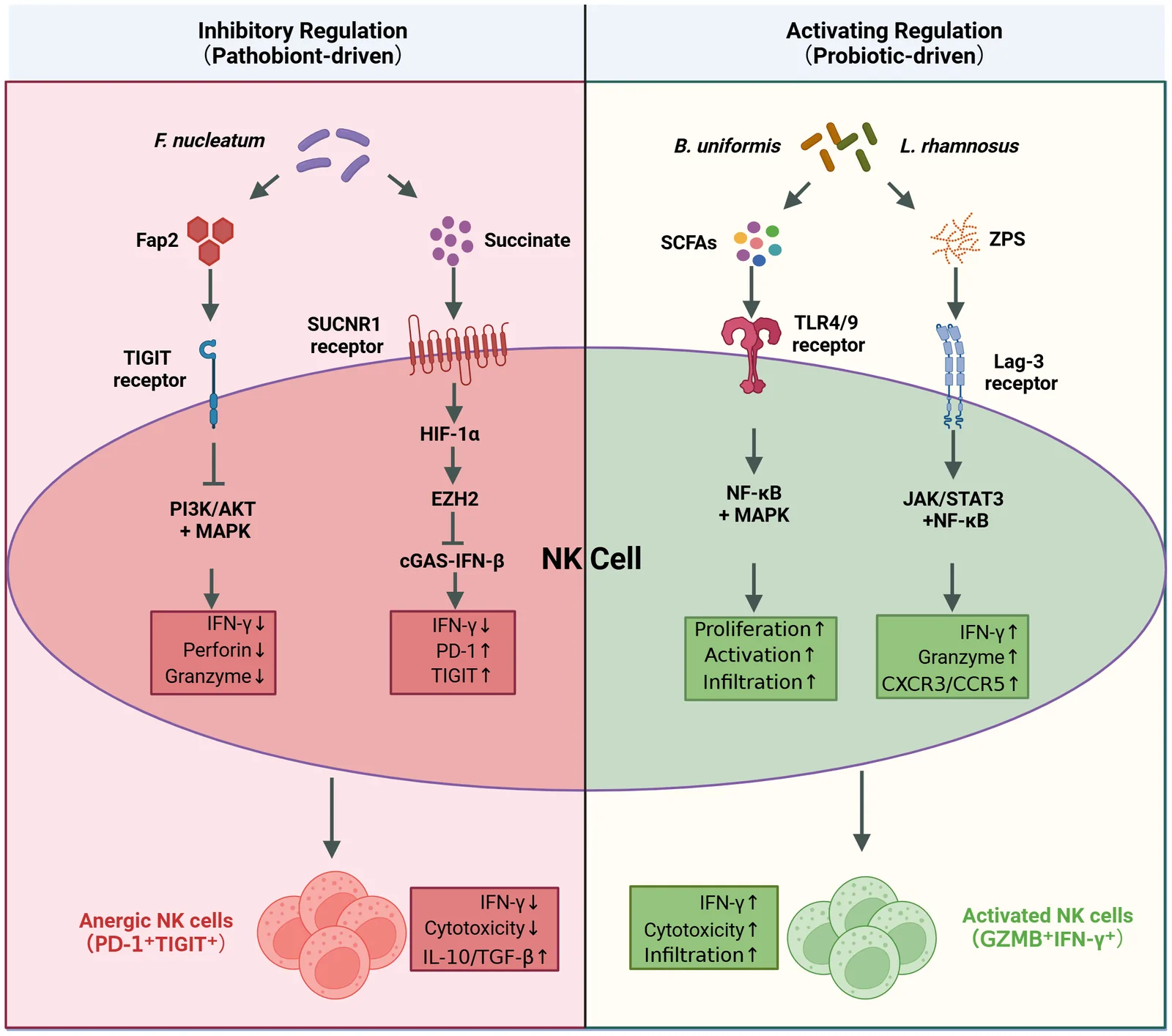
Bidirectional regulatory mechanisms of tumor microbiota on NK cells via surface proteins and metabolites. The left panel illustrates inhibitory pathways driven by pathogenic bacteria, primarily *F. nucleatum*. Its surface protein Fap2 binds to the inhibitory receptor TIGIT on NK cells, suppressing PI3K/AKT and MAPK signaling, leading to reduced granzyme B and perforin expression. Concurrently, *F. nucleatum*-derived succinate activates the SUCNR1–HIF-1α–EZH2 axis, which impairs IFN-γ production and upregulates inhibitory receptors (PD-1, TIGIT), promoting the transition toward dysfunctional NK cells (PD-1^+^TIGIT^+^). The right panel depicts activating pathways mediated by beneficial bacteria. *B. uniformis* capsular polysaccharide ZPS engages Lag-3, activating NF-κB and JAK/STAT3 signaling, thereby upregulating chemokine receptors (CXCR3, CCR5) and enhancing cytotoxic molecule expression (granzyme B, perforin). SCFAs derived from *L. rhamnosus* and *B. uniformis* activate TLR4/TLR9-dependent MAPK/NF-κB pathways, promoting NK cell proliferation, activation, and infiltration. These pathways collectively maintain the activated NK cell phenotype (GZMB^+^IFN-γ^+^).

At the bystander level, SCFAs maintain intestinal barrier integrity, limiting pathogenic bacterial invasion and indirectly preserving NK cell function (86). They also inhibit fatty acid synthase activity in CRC cells, counteracting tumor metabolic reprogramming and thereby increasing cancer cell susceptibility to NK cell‑mediated killing (87). At the human correlative level, fecal SCFA levels correlate positively with NK cell activation status in CRC patients (88). Furthermore, SCFA‑mediated NK cell activation enhances chemosensitivity in CRC, and this synergistic effect is more pronounced in patients carrying the wild‑type *TLR4* rs4986790 genotype (89). The bidirectional regulatory mechanisms of tumor microbiota on NK cells via surface proteins and metabolites are illustrated in **Figure 2**.

### 4.2 Memory-like NK cells: maintenance mechanisms and initial differentiation signals

In the CRC immune microenvironment, memory-like NK cells (CD45RO^+^CD69^+^) have attracted considerable attention due to their enhanced recall responses and capacity for long-term immune surveillance. However, progress in this field exhibits marked asymmetry: while the molecular mechanisms governing their long-term maintenance have been relatively well elucidated, the drivers of initial differentiation signals remain largely hypothetical, with a conspicuous evidentiary gap between the two. This section addresses the extant evidence for maintenance mechanisms and differentiation signals separately, and explicitly delineates the critical knowledge gaps pertaining to each.

#### 4.2.1 Maintenance pathway: the nicotinamide–SIRT1 axis

Among the currently defined regulatory pathways for memory-like NK cells, the most extensively investigated is the microbiota-derived metabolite-driven nicotinamide–SIRT1 axis. In contrast to pathways that enhance the functional capacity of activated NK cells, this axis primarily acts on already differentiated memory-like NK cells, supporting their long-term survival rather than inducing initial differentiation (90).

At the mechanistic level, nicotinamide, as a precursor of NAD^+^, exerts its critical effects through activation of the deacetylase SIRT1 (91–93). SIRT1 enhances mitochondrial biogenesis and oxidative phosphorylation in NK cells via deacetylation of FoxO1 and PGC-1α, thereby providing the metabolic basis for long-term survival of memory-like NK cells (44, 90, 94). Concurrently, SIRT1 attenuates NK cell exhaustion by deacetylating and inhibiting the transcriptional activity of NF-κB p65 (90, 91). At the gut microbiota level, the abundance of *A. muciniphila* correlates positively with the proportion of memory-like NK cells; however, this effect operates through the aforementioned nicotinamide metabolic pathway, representing indirect metabolic support rather than direct antigen-driven differentiation (43, 95). Short-chain fatty acids and nicotinamide both converge on mitochondrial fitness and metabolic homeostasis, providing a theoretical rationale for their synergistic action; nevertheless, direct evidence for their induction of memory-like NK cell differentiation in CRC remains lacking (90).

At the clinical translation level, nicotinamide has demonstrated efficacy in enhancing NK cell function and inducing remission in patients with non-Hodgkin lymphoma (90, 96), and nicotinamide-expanded NK products (NAM-NK) exhibit favorable *in vitro* persistence, lymphoid tissue homing capacity, and manufacturability. However, the aforementioned clinical evidence derives exclusively from hematological malignancies, and its applicability to CRC requires dedicated prospective cohort validation (97, 98). More importantly, this pathway does not address the fundamental question of how memory-like NK cells acquire their memory phenotype from a naïve state (71).

#### 4.2.2 Unresolved core question: three hypotheses for initial differentiation signals and their evidentiary boundaries

If the nicotinamide–SIRT1 pathway answers the question of how memory-like NK cells survive, then how they are generated constitutes the central unresolved scientific question in the field. Currently, three major non-mutually exclusive hypotheses have been proposed, though none has received direct causal validation in human CRC.

##### 4.2.2.1 Cytokine induction hypothesis: the IL-12/15/18 signaling axis

This hypothesis represents the most mechanistically advanced pathway to date. Multiple studies have demonstrated that NK cells, following brief co-stimulation with IL-12, IL-15, and IL-18, acquire durable memory-like functional enhancement, characterized by significantly elevated IFN-γ production, augmented cytotoxicity, and persistence for weeks to months after cytokine withdrawal (99, 100). These “cytokine-induced memory-like NK cells” have been validated in numerous preclinical studies and early-phase clinical trials, with mechanisms involving epigenetic remodeling of the IFN-γ locus, altered chromatin accessibility, and upregulation of transcription factors such as TCF7 and SELL (99, 101). In patients with acute myeloid leukemia, adoptive transfer of IL-12/15/18-pre-activated allogeneic NK cells has demonstrated clinical feasibility, with complete responses observed in preliminary studies (100).

However, the applicability of this hypothesis to CRC faces dual uncertainties. First, the aforementioned evidence derives predominantly from non-CRC models (e.g., murine lymphoma, melanoma, and human AML), and direct evidence that IL-12/15/18 within the CRC microenvironment suffice to trigger this differentiation program is lacking (99, 100). Second, the cellular sources, spatial localization and actual *in situ* concentrations of these cytokines in human primary CRC tissues remain without systematic histologic assessment.

##### 4.2.2.2 Tumor antigen-specificity hypothesis

This hypothesis posits that differentiation of memory-like NK cells requires an initial “sensitizing” signal from specific tumor antigens, mechanistically analogous to the antigen-driven process in adaptive T cells. Recent work in ovarian cancer has revealed that tumor-infiltrating adaptive NK (aNK) cells mount specific recall responses against autologous tumor antigens, a process dependent on the NKG2C–HLA-E axis (102). More critically, the investigators identified a non-classical peptide, APAPAPAPL, from ovarian tumors that binds HLA-E and effectively primes NK cells to mount enhanced memory responses against tumor cells (102, 103). These findings establish, for the first time in solid tumors, a causal chain from “tumor antigen peptide → NK cell memory differentiation”.

Nevertheless, this groundbreaking evidence is currently confined to ovarian cancer, and no tumor-derived HLA-E-restricted peptide has been identified as an inducer of NK cell memory differentiation in CRC. Furthermore, the “antigen specificity” of NK cells, in strict terms, remains a “HLA-E–NKG2C axis-dependent pattern recognition,” and whether it fundamentally differs from classical TCR-mediated antigen specificity at the molecular and signal transduction levels remains to be elucidated (102, 103).

##### 4.2.2.3 Microbial antigen or microbial community structure hypothesis

This hypothesis proposes that specific antigens or molecular patterns derived from the gut microbiota may provide the initial differentiation signal. Although *A. muciniphila* abundance correlates positively with memory-like NK cell proportions in CRC (104), and it can enhance NK–DC crosstalk through the STING agonist c-di-AMP (105), current evidence predominantly attributes the function of *A. muciniphila* to indirect modulation of the immunoregulatory microenvironment or metabolic maintenance effects, rather than direct induction of NK cell differentiation toward a memory-like phenotype. Additionally, *Alistipes putredinis* has been reported to increase peripheral blood memory CD8^+^ T cell and NK cell numbers (104), yet this association remains at the correlational level with no established causal relationship.

More fundamentally, no specific bacterial species or its products have been directly demonstrated to possess inductive activity for memory-like NK cell initial differentiation in CRC. Whether microbial antigens act indirectly through TLR signaling in concert with the cytokine network, or require cooperative support from specific microbial community structures, remains entirely unexplored.

Taken together, the three hypotheses are likely not mutually exclusive. The most plausible scenario is that initial differentiation of memory-like NK cells requires the synergistic action of specific microbial signals, local inflammatory cytokine networks, and tumor-derived antigen/HLA-E ligands, collectively constituting an integrated “induction–maintenance” signaling cascade. This integrative model conceptually resonates with the hierarchical frameworks of “trained immunity” and “NK cell adaptive” states, whereby distinct immunological experiences leave durable functional imprints on NK cells through epigenetic reprogramming and metabolic remodeling (101, 106). However, this model currently remains at the theoretical deductive stage, with no experimental data in CRC supporting its signal synergy relationships or temporal ordering.

#### 4.2.3 Critical evidence gaps

In summary, the molecular basis, spatial localization, and microbiota dependency of initial differentiation signals for memory-like NK cells remain among the most pressing unresolved questions in the field. All of the above hypotheses face two common evidentiary bottlenecks: (i) the vast majority of mechanistic inferences derive from non-CRC animal models or *in vitro* differentiation systems, and their reproducibility in human primary CRC tissues awaits validation (99–101); (ii) absence of direct causal evidence for phenotypic conversion—no lineage-tracing or *in situ* perturbation studies based on human CRC tissue have definitively tracked the complete trajectory of CD45RO^+^CD69^+^ NK cell conversion from a naïve state to a memory-like phenotype; and (iii) a void in spatial information—even if the three classes of signals do participate in differentiation induction, their spatial collaboration patterns within the CRC microenvironment, where they occur and by which cellular providers, lack any direct evidence with spatial resolution.

## 5 Regulatory networks of the microbiota–NK cell axis

Even with the above core axes defined, microbe–NK cell interactions exhibit substantial interindividual variability. This heterogeneity arises from three distinct but interacting sources: host genetics, recruitment and migration mechanisms, and cross‑kingdom microbial interactions (107, 108).

### 5.1 Modulation of axis efficiency by host genetics

Polymorphisms in immune receptor genes directly influence the efficiency of microbial signal transduction. For instance, the *TIGIT* rs7920644 polymorphism alters the binding affinity between Fap2 and TIGIT (12, 109); carriers of specific risk alleles may exhibit a predisposition toward elevated proportions of dysfunctional NK cells (56). Similarly, the *LAG-3* rs870849 GG genotype enhances ZPS‑mediated activation (26, 76), whereas the *SUCNR1* rs13079765 TT genotype increases susceptibility to succinate‑mediated inhibition (63).

Beyond these immune receptor genes, CRC susceptibility genes also shape this system (110). Patients carrying *APC* mutations exhibit increased *F. nucleatum* abundance and decreased *Bacteroides* abundance, correlating with reduced NK cell infiltration and enrichment of dysfunctional subsets (40). In contrast, patients with *MSH2* mutations, characteristic of microsatellite instability‑high (MSI‑H) tumors, display greater microbial diversity and higher proportions of activated NK cells (79, 110). Host immune‑related gene polymorphisms also contribute to this variability; for example, the *IFNG* rs2430561 TT genotype can amplify SCFA‑mediated NK activation (111), whereas the *IL10* rs1800896 AA genotype predisposes NK cells toward differentiation into the regulatory subset (112).

### 5.2 Recruitment and migration mechanisms

Tumor microbiota can influence NK cell recruitment and infiltration processes through both direct modulation of NK cell chemotactic capacity and indirect remodeling of the tumor microenvironment (78, 79).

Beneficial bacteria and their metabolites upregulate the expression of chemokine receptors, including CXCR3, CCR5, and CXCR6, on NK cells, thereby enhancing their responsiveness to tumor‑secreted chemokines such as CXCL10, CCL5, and CXCL16, and promoting NK cell migration from peripheral blood into tumor tissues (77, 79).

Pathogenic bacteria, by inducing the aggregation of immunosuppressive cells, construct immune barriers that obstruct NK cell infiltration pathways (58). *F. nucleatum* recruits MDSCs and M2‑type TAMs to the periphery of the tumor parenchyma, where these cells secrete suppressive factors such as TGF‑β and IL‑10, thereby inhibiting NK cell migration (113). In addition, lactate generated by pathogenic bacterial metabolism lowers the pH of the tumor microenvironment, inhibits actin polymerization in NK cells, impairs their motility, and consequently reduces their infiltration into the tumor core (67).

Furthermore, tumor microbiota can influence local NK cell recruitment in the gut by modulating intestinal mucosal barrier function. Beneficial bacteria help maintain mucosal barrier integrity, thereby promoting NK cell colonization within the intestinal mucosal layer (114, 115). In contrast, pathogenic bacteria disrupt the mucosal barrier, leading to disorganized NK cell distribution in the mucosal layer and impairing effective infiltration into tumor tissues (116).

### 5.3 Cross‑kingdom microbial synergy and antagonism

Bacteria do not act in isolation; fungi, viruses, and archaea modulate the microbiota–NK axis in ways that are only beginning to be understood (117). *C. albicans* can enhance *F. nucleatum*-mediated NK cell inhibition; although the precise molecular mechanism remains to be fully elucidated, their co-occurrence in the tumor microenvironment is associated with amplified immunosuppressive effects (60). Epstein–Barr virus (EBV) infection, through the LMP2A protein, enhances *Candida* colonization, further exacerbating NK dysfunction (118, 119). At the opposite end of the spectrum, *Methanobrevibacter* species produce methane, which improves intestinal redox balance, thereby enhancing *B. uniformis*‑derived ZPS secretion and subsequent NK activation (120). Currently, most microbiome analyses completely overlook fungi and archaea; whether integrating these kingdoms improves clinical prediction remains an open but important question (117, 121).

Interactions between fungi and viruses may further intensify immunosuppression. EBV infection, through LMP2A expression, enhances *C. albicans* colonization, thereby exacerbating NK cell dysfunction and promoting enrichment of the dysfunctional NK subset (118, 119).

In summary, the regulatory network of the microbiota–NK axis is co‑modulated by three interrelated dimensions: host genetics, recruitment and migration mechanisms, and cross‑kingdom interactions.

## 6 Microbiota-driven NK cell multicellular crosstalk network – from cascade initiation to immune remodeling

The microbiota orchestrates an antitumor microenvironment by triggering type I interferon (IFN-I) production from intratumoral monocytes, modulating macrophage polarization, and facilitating DC–NK cell crosstalk (122, 123). When microbiota homeostasis is disrupted, this monocyte–IFN-I–NK–DC cascade is interrupted, macrophages adopt a protumor phenotype, and the immune microenvironment shifts toward a suppressive direction (124). NK cells serve as a hub within this network, exhibiting both synergistic and antagonistic interactions with dendritic cells, T cells, and macrophages, with microbial signals acting as a critical variable that determines the direction and intensity of these interactions (31).

### 6.1 Microbiota-driven NK–DC synergy axis: from IFN-I cascade to positive feedback loop

An intact microbiota induces IFN-I production from intratumoral monocytes via STING agonists, which in turn modulates macrophage polarization and DC–NK cell crosstalk to establish an antitumor microenvironment (122, 123). A high-fiber diet improves immune checkpoint blockade efficacy by engaging this IFN-I–NK–DC axis (122). An additional mechanism by which the gut microbiota enhances antitumor immunity involves accelerating the maturation and migration of CD103^+^CD11b^-^ conventional DCs (cDCs), thereby increasing the number of CD8^+^ T cells responding to tumor antigens (125).

At the level of DC–NK bidirectional crosstalk, DCs influence NK cell survival and optimal activation, whereas NK cells stimulate DC survival, maturation, and tumor infiltration through the release of soluble factors (126). NK cells promote the abundance of stimulatory dendritic cells (SDCs) within the tumor microenvironment through FLT3L secretion, and this axis correlates positively with patient responses to anti-PD-1 immunotherapy and overall survival (31). Following NK cell-mediated DC activation, DCs secrete IL-12, which enhances NK cell IFN-γ production, thereby establishing a positive feedback loop. Indole-producing microbiota can further potentiate this immune response and may serve as an adjunct to NK cell-based immunotherapy (127).

### 6.2 NK–myeloid cell antagonistic interactions under microbial dysbiosis

When the microbiota becomes imbalanced, the interactions between NK cells and myeloid cells shift from synergistic to antagonistic. Tumor-associated NK cells drive monocyte and neutrophil differentiation into MDSCs through IL-6 secretion, increasing S100A8/9 and arginase-1 expression and suppressing T cell responses (128). MDSCs not only possess intrinsic suppressive functions but also amplify the immunosuppressive activities of macrophages and DCs through crosstalk (129). In a germ-free setting, the tumor microenvironment favors a protumor macrophage phenotype, and the absence of microbial signals precludes proper initiation of this cascade (122). Myeloid cells inhibit NK activity through downregulation of NK receptors and depletion of nutritional factors (35). Butyrate, a short-chain fatty acid, abrogates the antitumor effects enhanced by vancomycin (130), suggesting a complex role for specific microbial metabolites in myeloid-mediated immunosuppression.

In terms of remodeling direction, an intact gut microbiota combined with immune checkpoint inhibitors (ICIs) can reprogram macrophages from protumor Spp1^+^ TAMs toward Cd74^+^ TAMs with antigen-presenting capacity (131). This process involves the γδ T cell–APC–CD8^+^ T cell axis: microbiota and ICIs enhance CD40lg expression on γδ T cells, which activates APCs through CD40–CD40L NF-κB signaling (131).

The key antagonistic interactions between NK cells and myeloid cells under microbial dysbiosis are summarized in **Table 2**.

**Table 2:** Antagonistic NK–myeloid cell interactions under microbial dysbiosis.

Interaction type	Microbial influence	Antagonistic mechanism	Ref.
NK–MDSC	Microbial dysbiosis drives NK cell reprogramming	Tumor-experienced NK cells drive MDSC expansion via the IL-6/STAT3 axis, suppressing T cells	(128)
NK–Macrophage	Germ-free environment skews TME toward protumor macrophages.	MDSCs suppress NK function through release of PGE_2_, TGF-β, etc.	(122, 132)
DC–NK–Macrophage	SCFAs (butyrate) abrogate vancomycin-enhanced antitumor effects	Myeloid cells suppress NK activity via downregulation of NK receptors and nutrient depletion	(35, 130)

### 6.3 The dual network of microbiota–NK–T cell complementarity and antagonism

NK cells and CD8^+^ T cells play complementary roles in defense against pathogens and cancer, with NK cells providing innate cytotoxicity and T cells mediating adaptive immunity (132). T cell-derived IL-2 activates NK cells, while NK cells recruit DCs to tumors and enhance CD8^+^ T cell responses (133). In terms of dual-target synergy, MICA/MICB cancer vaccines can simultaneously engage T cells and NK cells in coordinated attack, demonstrating efficacy in mice and rhesus macaques (134). NK cells activate macrophages and T cells through IFN-γ secretion; however, cancer cells treated with IFN-γ become resistant to NK killing, suggesting that the primary purpose of this signal may be to recruit other immune cells rather than direct cytotoxicity (127).

Nevertheless, under microbial dysbiosis conditions, *F. nucleatum* promotes MDSC/Treg enrichment through the TLR4–NF-κB and Fap2–TIGIT pathways, indirectly impairing NK–T cell antitumor immunity (135). Tumor-associated NK cells undergo functional reprogramming toward a regulatory phenotype, suppressing CD8^+^ T cell activity through cytokine competition and immunoediting, leading to immune checkpoint blockade resistance (134). Beneficial commensals such as *Lactobacillus* and *Bifidobacterium* serve as probiotics that enhance NK cell activity and function, promoting inflammatory cytokine expression (123).

### 6.4 An integrated view of microbiota-mediated regulation of the multicellular crosstalk network

**Figure 3:**
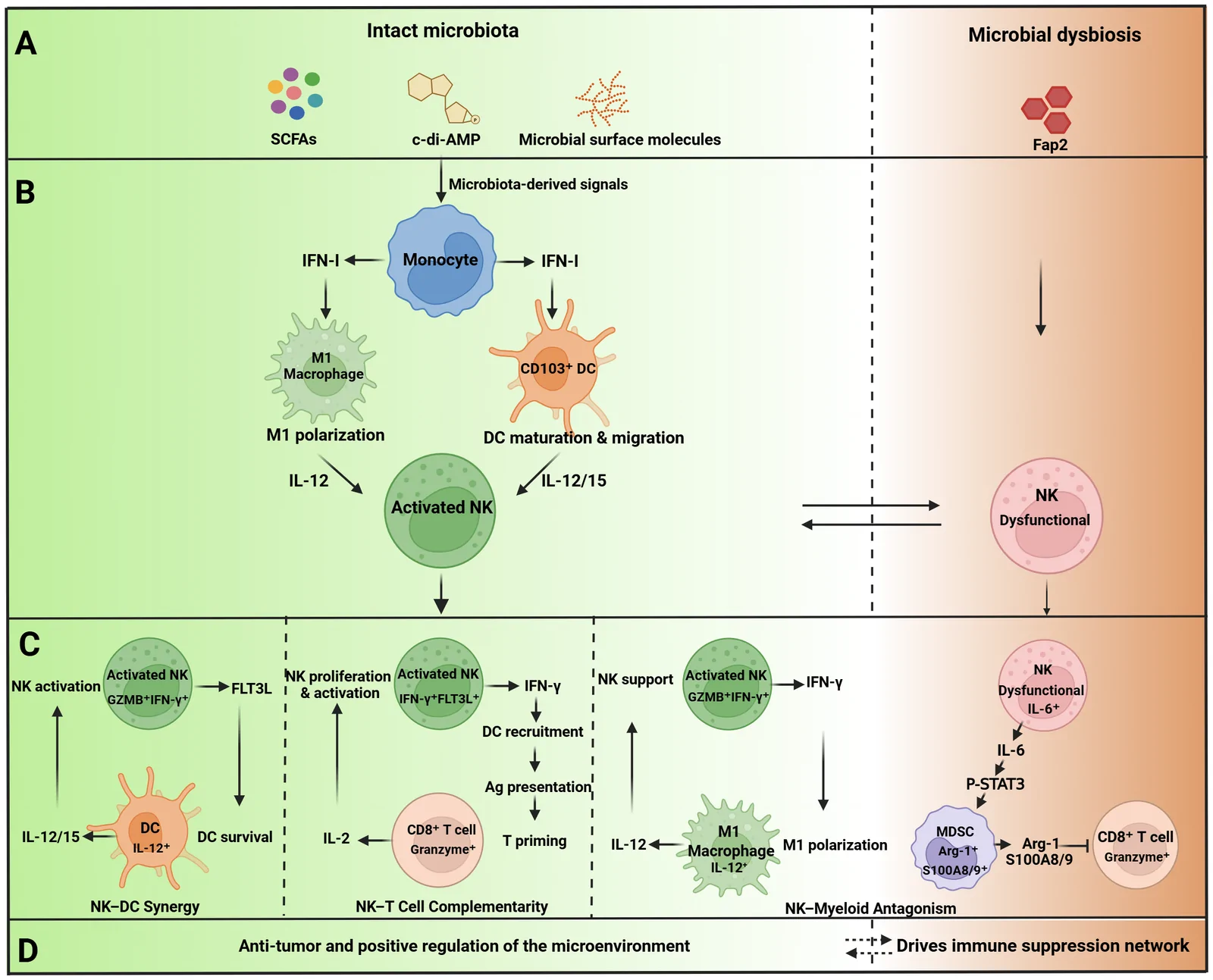
Microbiota-driven regulation of the NK cell multicellular crosstalk network in colorectal cancer. This figure presents a hierarchical cascade model depicting how gut microbiota-derived signals reshape the tumor microenvironment and orchestrate natural killer cell-mediated multicellular interactions, ultimately determining the balance between antitumor immunity and immunosuppression. **(A)** Microbial signals and microbiota status. The left panel (green) represents microbiota-intact states with beneficial signals (STING agonists, SCFAs). The right panel (red) represents dysbiosis with pathogenic signals (F. nucleatum surface molecules such as Fap2, and other metabolites). **(B)** Initiation hub – monocyte/macrophage and DC–NK crosstalk. Under microbiota-intact conditions, STING agonists induce IFN-I production in monocytes, driving M1 macrophage polarization and CD103^+^ DC maturation/migration, followed by IL-12/15-mediated NK activation. Under dysbiosis, pathogenic signals directly promote the generation of dysfunctional NK cells. **(C)** NK-mediated effector network. Three axes are depicted: Left: NK–DC synergy. Activated NK cells (GZMB^+^IFN-γ^+^) and DCs (IL-12^+^) form a positive feedback loop via FLT3L and IL-12/15 signaling (circular arrow); Middle: NK–T cell complementarity. NK cells (IFN-γ^+^FLT3L^+^) and CD8^+^ T cells (Granzyme^+^) engage in bidirectional crosstalk through IFN-γ and IL-2; Right: NK–myeloid antagonism (double-edged). Under microbiota-intact conditions (upper, green), NK cells synergize with M1 macrophages via IFN-γ/IL-12. Under dysbiosis (lower, red), dysfunctional NK cells secrete IL-6, which activates STAT3 signaling (p-STAT3^+^) in MDSCs, driving their expansion and subsequent CD8^+^ T-cell suppression via S100A8/9 and Arg-1. **(D)** Microbiota-guided polarity. Microbiota-intact and dysbiotic states lead to pro-inflammatory/activating and anti-inflammatory/suppressive networks, respectively, with dynamic transition indicated by the dashed arrow.

Synthesizing the three interaction axes detailed above (Sections 6.1-6.3), we propose a hierarchical cascade model to conceptualize how the microbiota regulates the NK cell multicellular crosstalk network (**Figure 3**). This integrated framework progresses through three functional layers: microbial signal input (Layer 1), immune initiation (Layer 2), and NK effector network (Layer 3).

Layer 1 categorizes microbial inputs based on community status. Under microbiota-intact conditions, beneficial signals (e.g., STING agonists and SCFAs) predominate; under dysbiosis, pathogenic signals (e.g., F. nucleatum-derived Fap2 and metabolites) prevail, setting the upstream direction of the cascade.

Layer 2 serves as the initiation hub. Intact microbiota triggers a STING–IFN-I–M1/DC–IL-12 axis that licenses NK activation, whereas dysbiosis directly promotes the emergence of dysfunctional NK cells. This functional dichotomy at the initiation step determines the subsequent effector network polarity.

Layer 3 constitutes the NK effector network, comprising three integrated axes: (i) NK–DC synergy through FLT3L and IL-12/15 forms a positive feedback loop; (ii) NK–T cell complementarity via IFN-γ and IL-2 bridges innate and adaptive immunity; and (iii) NK–myeloid antagonism exhibits context-dependent duality, switching from M1-supported activation under intact microbiota to MDSC-mediated suppression under dysbiosis.

The core principle emerging from this framework is that the microbiota acts as a rheostat rather than a passive bystander. When intact, it sustains a pro-inflammatory/activating network that amplifies antitumor immunity; when dysbiotic, it shifts the system toward an anti-inflammatory/suppressive network that fosters immune evasion. This polarity is dynamic and reversible, providing a mechanistic rationale for microbiota-targeted interventions to reprogram the TME. The microbiota, as an underappreciated component of the tumor microenvironment, modulates NK cell crosstalk with DCs, T cells, and myeloid cells through this multidimensional network, offering potential nodes for improving cancer immunotherapy (134).

## 7 Spatial organization of the microbiota–NK cell axis

Beyond interindividual regulatory differences, spatial location represents another critical determinant of microbial–NK functional outcomes (23, 24). This section focuses on local spatial organization and metastatic site specificity.

### 7.1 Methodological caveat

It should be noted that current spatial transcriptomics data are derived primarily from single‑center studies with limited sample sizes, typically including 50 or fewer patients. Consequently, their generalizability requires independent validation in multicenter, large‑cohort settings. The spatial distance metrics cited herein should be regarded as exploratory observations rather than established spatial biomarker thresholds (24, 136). Furthermore, different studies employ varied capture technologies, such as 10x Visium and Multiplexed error‑robust fluorescence *in situ* hybridization (MERFISH) (137), along with disparate tissue processing methods and analytical pipelines; therefore, direct cross‑study comparisons of spatial distance metrics should be interpreted with caution.

### 7.2 From spatial correlation to spatial causality: mechanisms linking proximity to NK cell dysfunction

Although spatial transcriptomics has convincingly demonstrated the co‑localization of *F. nucleatum* with dysfunctional NK cells and of *B. uniformis* with activated NK cells, the critical unresolved question is whether physical proximity directly drives functional changes or merely reflects a pre‑existing immune compartmentalization. Emerging evidence is beginning to address this causality gap by revealing that spatial proximity creates distinct microenvironmental niches in which locally concentrated microbial products exert direct, distance‑dependent effects on neighboring NK cells.

One mechanistic link is provided by the establishment of concentration gradients of bacterial metabolites. For instance, succinate produced by *F. nucleatum* within the tumor parenchyma is subject to rapid local consumption and clearance, generating a steep concentration gradient that is highest within 50 µm of the bacterial source. This localized accumulation selectively activates the SUCNR1–HIF‑1α–EZH2 axis in adjacent NK cells in a paracrine manner, impairing IFN‑γ production and upregulating inhibitory receptors, while NK cells located beyond this gradient remain relatively spared (62, 74). Similarly, lactate generated by the metabolic activity of pathogenic bacteria lowers local pH and inhibits actin polymerization in neighboring NK cells, impairing their motility and cytotoxic function in a spatially restricted manner (67).

Beyond metabolite gradients, direct cell–cell contact mechanisms also confer spatial specificity. The Fap2–TIGIT interaction requires physical engagement between *F. nucleatum* and NK cells, rendering this inhibitory signal inherently distance‑dependent (12). Notably, spatial transcriptomic data suggest that NK cells located within 50 µm of *F. nucleatum*‑colonized tumor cells exhibit a 60‑70% reduction in GZMB expression compared with those at greater distances, a phenomenon that has been termed the “inhibitory zone” (24, 137). This distance‑effect relationship strongly supports a causal interpretation: the physical proximity to the microbial source, rather than merely the overall microbial abundance, determines the intensity of the inhibitory signal.

In parallel, the perivascular activation center represents a spatially privileged niche where beneficial microbes and activated NK cells co‑localize. The enrichment of *B. uniformis* and its ZPS in perivascular regions establishes a local activating gradient that enhances trans‑endothelial migration and sustains NK cell activation through the LAG‑3–NF‑κB axis (26, 78). This spatial arrangement suggests that perivascular niches function as immune “launch pads,” from which activated NK cells can infiltrate the tumor parenchyma, provided that the inhibitory barrier established by *F. nucleatum* does not impede their path.

Taken together, these mechanistic insights support the view that the spatial distance between NK cells and specific microbes is not merely a correlative marker but a functional determinant of NK cell fate. However, it must be emphasized that most of these mechanistic interpretations are derived from preclinical models and *in vitro* systems; direct causal validation in human CRC tissues, for example, through spatially resolved perturbation experiments or longitudinal sampling, remains a critical knowledge gap. Furthermore, these spatial gradients and contact-dependent mechanisms are not fixed; they evolve dynamically as tumors progress and metastatic dissemination occurs, a dimension addressed in the following section.

### 7.3 Dynamic evolution of spatial immune hubs during tumor progression

Importantly, the spatial patterns described above are not static snapshots but undergo dynamic remodeling as CRC progresses. This temporal dimension is essential for understanding when and how the microbiota–NK spatial architecture becomes established and whether it can be therapeutically reversed.

In early‑stage CRC, the spatial distribution of microbes and NK cells is relatively homogeneous. *F. nucleatum* colonization is minimal, and NK cell density is comparable between the tumor parenchyma and the surrounding stroma, with most NK cells exhibiting an activated phenotype (136). During this phase, the gut–tumor axis delivers systemic short‑chain fatty acids and other beneficial metabolites that support global immune surveillance, and no discrete inhibitory or activating zones are apparent (138).

As tumors advance to late‑stage CRC, a process of spatial selection emerges. The hypoxic, immunoprivileged environment of the tumor parenchyma favors the expansion of *F. nucleatum*, which is relatively resistant to immune clearance and benefits from reduced immune surveillance within the tumor core. This parenchymal enrichment creates the inhibitory zone, which progressively expands outward over time. Concomitantly, *B. uniformis* and other beneficial commensals are gradually displaced from the parenchyma to the perivascular and stromal areas, driven by differences in oxygen tension, nutrient availability, and local immune pressure. This displacement paradoxically establishes the perivascular activation center, as the remaining *B. uniformis* niches serve as reservoirs for maintaining activated NK cells, albeit confined to the stromal compartment (24, 137).

This dynamic perspective is supported by spatial transcriptomic data showing that the width of the inhibitory zone correlates with disease stage**;** in advanced CRC, the zone extends further into the stroma, accompanied by a progressive decline in total NK cell density within the parenchyma and an enrichment of the dysfunctional subset (58). Notably, this spatial remodeling may also be influenced by cross‑kingdom interactions. For example, *C. albicans* colonization, which becomes more prevalent in advanced tumors, is associated with enhanced *F. nucleatum*-mediated NK cell inhibition within the established inhibitory zone, although the precise molecular mechanism remains to be fully elucidated (60). Similarly, the presence of *Candida* may further displace beneficial bacteria from the parenchyma through competitive exclusion, accelerating the spatial polarization of the immune landscape.

The clinical relevance of these dynamic changes is underscored by preliminary evidence that therapeutic interventions can remodel spatial architecture. In preclinical models, *F. nucleatum*‑targeting phage therapy not only reduces bacterial load but also shrinks the inhibitory zone, allowing activated NK cells from perivascular niches to infiltrate previously excluded parenchymal regions (139). However, whether such spatial remodeling translates into durable clinical responses in CRC patients remains to be established through prospective, time‑resolved studies that capture the evolution of spatial immune hubs before, during, and after therapy.

**Figure 4:**
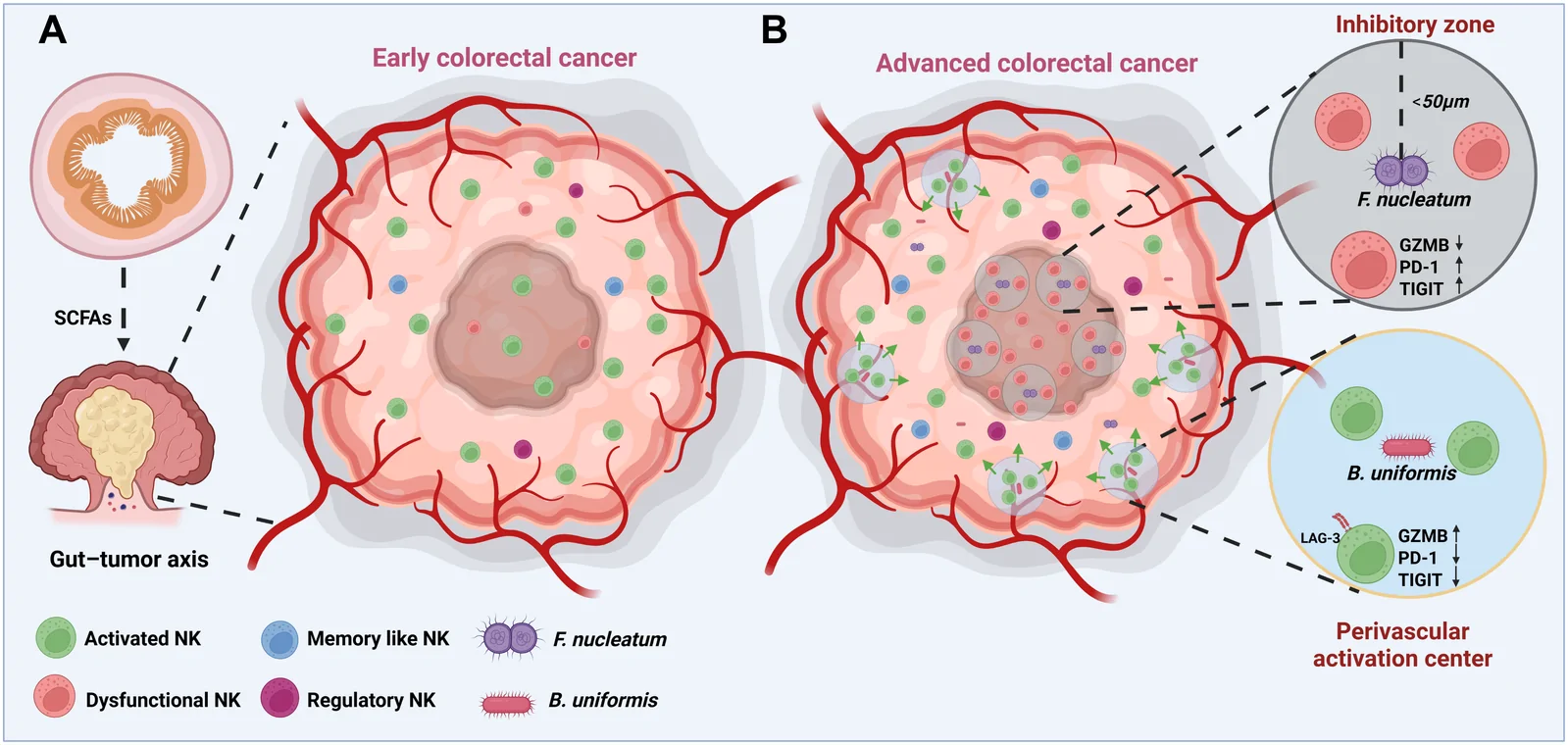
Spatial organization of the microbiota–NK cell axis in colorectal cancer: the “50 µm inhibitory zone” and “perivascular activation center” model. This figure illustrates the spatial dichotomy of NK cell functional states in relation to microbial localization within the tumor microenvironment. **(A)** Early-stage colorectal cancer – balanced distribution. In early-stage tumors, *F. nucleatum* colonization is minimal, and NK cells are evenly distributed across the tumor parenchyma, stroma, and perivascular regions, with the activated phenotype predominating. The gut–tumor axis delivers systemic short-chain fatty acids (SCFAs) that support local immune surveillance. **(B)** Advanced colorectal cancer – spatially compartmentalized immune landscape. In advanced tumors, *F. nucleatum* becomes enriched within the tumor parenchyma, creating a <50 µm inhibitory zone in which neighboring NK cells exhibit a dysfunctional phenotype characterized by low GZMB and elevated PD-1/TIGIT expression. In contrast, perivascular areas serve as activation centers, where *B. uniformis* and LAG-3^+^ activated NK cells co-localize, facilitating trans-endothelial migration and maintaining robust antitumor activity. The tumor stroma harbors a mixed population of activated, memory-like, and regulatory NK cells, preserving immune surveillance functions.

Collectively, these observations challenge the notion of a static, one‑dimensional view of microbe–NK interactions and underscore the need for temporal and spatial integration when designing microbiota‑targeted interventions. Longitudinal spatial sampling—for example, through paired primary and recurrent tumor biopsies or sequential biopsies in metastatic disease—is urgently needed to distinguish whether the observed spatial architecture is a cause or a consequence of immune evasion, and to identify the optimal therapeutic window for intervention (**Figure 4**).

### 7.4 Metastatic site specificity: differences in liver, lung, and bone metastases

This spatial heterogeneity extends to metastatic lesions and carries important clinical implications. In CRC liver metastases, *F. nucleatum* expands from the parenchyma to the invasive margin, coinciding with the accumulation of dysfunctional NK cells. Mechanistically, this margin-specific enrichment may reflect the liver’s unique vascular architecture and immunotolerant milieu, which synergize with *F. nucleatum*-derived inhibitory signals to promote local NK dysfunction (58, 140, 141). In lung metastases, a distinct pathogen, *Klebsiella pneumoniae* (*K. pneumoniae*), co-enriches with dysfunctional NK cells, suggesting site‑specific microbe–NK regulation that may operate through Toll-like receptor or C-type lectin receptor pathways distinct from the Fap2–TIGIT axis (136, 141). Although studies on bone metastases remain limited, the bone marrow’s role as a primary site of NK cell development and reservoir for mature NK cells raises the intriguing but as-yet-unexplored possibility that unique microbial–immune interactions may operate in this niche, warranting future investigation (10, 136).

Critically, emerging evidence indicates that these site-specific microbial–NK associations are not static but subject to dynamic spatial remodeling over the course of metastatic progression. Longitudinal sampling of paired primary tumors and their corresponding metastases suggests that the spatial architecture of the microbiota–NK axis—including the width of the inhibitory zone and the integrity of perivascular activation centers—may evolve as tumors adapt to new microenvironmental niches (58, 141). This dynamic evolution has direct therapeutic implications: preclinical studies have shown that *F. nucleatum*-targeting phage therapy can reduce the inhibitory zone and restore NK cell infiltration in liver metastasis models, suggesting that spatial remodeling is achievable through intervention (139). However, whether such remodeling translates into durable clinical responses, and whether the distinct microbial drivers in lung metastases require fundamentally different therapeutic approaches, remain open questions that necessitate site-stratified prospective trials (88).

Collectively, these observations challenge the notion of a “one‑size‑fits‑all” microbiota‑targeting strategy and underscore the need for differentiated intervention protocols based on metastatic site, with attention to the evolving spatial landscape over time (37, 142). **Table 3** consolidates the core spatial findings and their current evidence levels.

**Table 3:** Summary of core spatial organization findings.

Spatial phenomenon	Description	Clinical implication	Ref.
50 µm inhibitory zone	NK cells within <50 µm of *F. nucleatum* colonized cells show 60-70% reduction in GZMB	Suggests spatial proximity determines inhibition strength; mediated by metabolite gradients and Fap2–TIGIT contact	(24, 137)
Perivascular activation center	*B. uniformis* co localizes with activated NK cells around vessels	May represent a niche for maintaining immune surveillance; serves as immune “launch pad”	(26, 78)
Dynamic spatial remodeling	Inhibitory zone expands with tumor progression; beneficial bacteria displaced to stroma	Intervention timing is critical; spatial architecture may be reversible with therapy	(24, 58, 137)
Liver metastasis specificity	*F. nucleatum* enriched at the invasive margin	Site specific interventions should be considered	(58, 140, 141)
Lung metastasis specificity	*K. pneumoniae* co-enriches with dysfunctional NK cells	Mechanisms may differ by metastatic site	(136, 141)
Gut-tumor axis	SCFAs and other metabolites reach distant tumors via circulation	Oral interventions have a theoretical basis for modulating distant sites	(136)

## 8 Clinical translation: two hypothesis‑driven precision pathways

Translating these mechanistic and spatial insights into testable clinical hypotheses requires a fundamental departure from “one‑size‑fits‑all” strategies (27). This section proposes a conceptual stratification framework for future clinical validation. **Table 4** summarizes the major interventions, their targeted axes, development stages, and evidence levels.

**Table 4:** Clinical development stage and evidence level of major interventions in this framework.

Intervention	Targeted axis/mechanism	Clinical development stage	Remarks	Ref.
TIGIT blockade	Fap2-TIGIT axis	Phase III (multiple solid tumors)	Tiragolumab available; more CRC specific data needed	(56, 139)
Nicotinamide supplementation	Nicotinamide-SIRT1 axis	Marketed (nutritional supplement); clinical data in NHL	Prospective CRC trials needed	(90)
*F. nucleatum* targeting phage	Fap2-TIGIT axis	Early phase (I/II)	Preliminary objective response rate (ORR) 42%, small sample	(88, 143)
SUCNR1 antagonists	Succinate-SUCNR1 axis	Preclinical (animal models)	No CRC clinical trials yet	(62, 75)
ZPS supplementation/*B. uniformis*	ZPS-LAG-3 axis	Preclinical (animal models)	Mechanistic research stage	(26)
SCFA precursors/analogues	SCFA-TLR axis	Partially marketed (supplements); exploratory in CRC	Rigorous RCTs needed	(82, 83)
Fecal microbiota transplantation (FMT)	Multi axis remodeling	Early clinical exploration (oncology)	Donor Human leukocyte antigen (HLA) matching not standard	(88, 144)
LAG 3 agonists	ZPS-LAG-3 axis	Not in any clinical development stage	Currently conceptual; molecular design is major bottleneck	(26, 76, 142)

The strategies outlined below are deliberately anchored on the three well-characterized NK subsets (activated, dysfunctional, and memory-like) for which defined microbial pathways and mechanistic evidence exist. The regulatory-like state, lacking validated microbial drivers (see Section 2.4), is not incorporated into this translational framework.

Important Disclaimer: The following pathways are conceptual hypothesis frameworks for research design, not validated clinical protocols. All interventions require prospective trial validation before clinical application.

### 8.1 Pathway A: enrichment strategy (immunologically “hot” patients)

For immunologically “hot” patients, characterized by high *B. uniformis* abundance (53), low *F. nucleatum* abundance (58), elevated intratumoral proportions of LAG-3^+^ activated NK cells (26), and low proportions of TIGIT^+^ dysfunctional subsets (56), an enrichment strategy is prioritized. The core rationale is to enhance the existing antitumor immune response.

The conceptual intervention package for this pathway includes multiple components: supplementation with *B. uniformis*‑derived polysaccharide (ZPS) or live bacterial formulations, aimed at activating the LAG-3^+^ NK cell axis—preclinical models have shown enhanced NK cell infiltration and increased proportions of activated subsets (26, 53); concurrent administration of short‑chain fatty acid precursors or analogues, such as acetate, propionate, and butyrate, to potentiate NK cell activity and sustain activation through the TLR–MAPK pathway, with exploratory clinical data available (82, 83); additionally, leveraging the unique activating effect of LAG-3 on NK cells, LAG-3 agonists could theoretically enhance this pathway, although it must be emphasized that no LAG-3 agonist has yet entered clinical development, and molecular design and pharmacology remain major bottlenecks (88, 142, 145); as an optional supplementary approach, adoptively transferred NK cells in *B. uniformis*‑preconditioned models exhibit enhanced tumor infiltration and persistence (146, 147). However, key uncertainties of this strategy include the incompletely elucidated molecular mechanism underlying the LAG-3 paradox and the general lack of direct human *in vivo* validation for the above interventions, both of which constrain clinical translation (26, 76).

### 8.2 Pathway B: remodeling strategy (immunologically “cold” MSS patients)

For immunologically “cold” MSS patients, characterized by high *F. nucleatum* abundance (40, 58), elevated succinate levels (62), dominance of TIGIT^+^ dysfunctional NK cells (56), and often carrying risk‑associated *TIGIT* or *SUCNR1* alleles (12, 63, 109), a remodeling strategy should be adopted. Its core logic is to first relieve immunosuppression and subsequently introduce activating signals. This intervention sequence is mandatory: first, *F. nucleatum*‑targeting phage therapy to rapidly reduce bacterial load and eliminate the continuous source of Fap2 protein and succinate; this strategy has entered phase I/II clinical exploration (88, 143). Subsequently, SUCNR1 antagonists to block residual succinate‑mediated signaling, currently in preclinical validation (62, 75). On this basis, TIGIT blockade to directly reverse NK cell exhaustion; this target has phase III cross‑cancer data (56, 139). Finally, after relieving the inhibitory microenvironment, supplement with SCFAs or perform FMT to introduce positive activating signals and promote NK cell functional recovery (88, 144). However, this pathway faces multiple critical uncertainties. First, the causal direction remains unclear; the temporal sequence between high *F. nucleatum* abundance and NK dysfunction requires time‑resolved studies for confirmation (12, 58). Second, the safety of combinatorial regimens has not been systematically evaluated, and sequential use of multi‑mechanism drugs may yield unforeseeable additive toxicities. Moreover, donor selection criteria for FMT remain unstandardized, and the reconstitution efficacy of different donor microbiota after relief of suppression may vary considerably. All these factors constrain the clinical translation of the remodeling strategy (148). **Table 5** compares the two pathways across six key dimensions to highlight their distinct rationales and knowledge gaps.

**Table 5:** Comparison of the two precision pathways.

Dimension	Pathway A: enrichment strategy	Pathway B: remodeling strategy
Target population	High *B. uniformis*, low *F. nucleatum*, LAG-3^+^ activated NK dominant	High *F. nucleatum*, high succinate, TIGIT^+^ dysfunctional NK dominant
Core logic	Enhance pre-existing antitumor immunity	Relieve suppression first, then introduce activation
Intervention sequence	Concurrent or sequential	Mandatory sequence: Phage → SUCNR1 antagonist → TIGIT blockade → SCFAs/FMT
Key evidence	ZPS activates LAG-3-NK axis (animal); *B. uniformis* correlates with immunotherapy response (human)	Targeted phage reduces *F. nucleatum* load (early clinical); SUCNR1 antagonist reverses dysfunction (animal); TIGIT blockade restores NK function (animal/cross cancer)
Clinical development stage	ZPS/LAG-3- agonist: preclinical; SCFAs: exploratory	Phage: phase I/II; SUCNR1 antagonist: preclinical; TIGIT blockade: phase III (other solid tumors)
Evidence level	Low-moderate	Moderate (TIGIT blockade has cross cancer data)
Core uncertainties	LAG-3 paradox unresolved; human validation lacking	Causal direction unclear; combination safety unknown

Dynamic adjustment: The two pathways are not mutually exclusive. Patients who initiate the remodeling pathway and successfully clear *F. nucleatum* can transition to the enrichment pathway for maintenance (88). Conversely, patients on the enrichment pathway who develop secondary *F. nucleatum* colonization may require temporary remodeling intervention (58). Such dynamic, adaptive strategies should be guided by continuous fecal and biopsy monitoring (53, 79).

## 9 Special implications for MSS CRC

The framework described above is of particular relevance for patients with refractory MSS disease. This assessment is grounded in the fundamental differences between MSS and MSI-H tumors with respect to tumor microenvironment composition, immune evasion mechanisms, and therapeutic responses (110).

### 9.1 MSS versus MSI-H: multidimensional biological and clinical distinctions that dictate divergent therapeutic strategies

MSS and MSI-H tumors differ fundamentally across four interrelated dimensions that collectively shape the microbiota–NK cell axis and dictate distinct therapeutic approaches (27, 59, 149).

#### 9.1.1 Mutational burden and neoantigen landscape

MSI-H tumors, owing to their high mutation frequency, generate abundant neoantigens and typically exhibit an “immune-inflamed” tumor microenvironment characterized by robust CD8^+^ T cell and NK cell infiltration, along with high expression of checkpoint molecules such as PD-1, PD-L1, and CTLA-4. Consequently, immune checkpoint inhibitors (ICIs) demonstrate marked efficacy in these tumors, with objective response rates of approximately 30–50% (27, 149). In contrast, MSS tumors harbor low mutational burden and scarce neoantigens, manifesting as an “immune-desert” or “immune-excluded” tumor microenvironment (110). Accordingly, PD-1/PD-L1 monotherapy yields objective response rates of only 0–5% in MSS (27, 149). This stark contrast constitutes a fundamental therapeutic challenge: in MSS, the absence of robust T cell attack creates a vacuum that innate immunity—particularly NK cells—must fill, yet the MSS microenvironment actively suppresses NK cell function through multiple converging mechanisms.

#### 9.1.2 Spatial immune architecture and NK cell infiltration patterns

MSS tumors characteristically exhibit hallmark features of physical immune restriction—immune cells, including NK cells, are largely confined to the tumor stroma with limited penetration into the tumor parenchyma (116). This spatial exclusion is far less prominent in MSI-H tumors, where immune cells are more evenly distributed across both stromal and parenchymal compartments (28). Consequently, the spatial patterns described in this framework—namely, the “50-μm inhibitory zone” surrounding *F. nucleatum*-colonized tumor cells and the “perivascular activation centers” where *B. uniformis* colocalizes with activated NK cells (136, 150)—may be amplified in MSS tumors. This spatial organization creates physical barriers that not only restrict NK cell access to tumor cells but also concentrate the immunosuppressive effects of *F. nucleatum* within the parenchymal compartment, while preserving a reservoir of activatable NK cells within perivascular niches that remain therapeutically accessible. The clinical implication is straightforward: spatial reprogramming strategies—such as redirecting beneficial bacteria to perivascular niches via fecal microbiota transplantation, or enhancing NK cell chemotaxis through microbial metabolites—may yield greater efficacy in MSS than in MSI-H, where immune cells already have unimpeded access to the tumor parenchyma.

#### 9.1.3 Metabolic microenvironment

MSS tumors, particularly those with high *F. nucleatum* abundance, exhibit increased accumulation of succinate and lactate within the tumor microenvironment (62, 67). Succinate derived from *F. nucleatum* metabolism activates the SUCNR1–HIF-1α–EZH2 axis on NK cells, promoting their dysfunction (61, 62). Concurrently, lactate reduces local pH and suppresses PI3K/AKT signaling in NK cells, further impairing their cytotoxic capacity (67, 68). This metabolically hostile environment is less pronounced in MSI-H tumors, which maintain a more permissive metabolic profile that better supports immune effector cell function. The therapeutic corollary is that metabolic interventions, such as blocking succinate signaling with SUCNR1 antagonists or counteracting lactate-mediated acidosis with alkalinizing agents, represent rational adjunct strategies specifically tailored to MSS, given that metabolite-driven immunosuppression is a dominant feature of this subtype.

#### 9.1.4 Microbial colonization profiles

MSS and MSI-H tumors exhibit distinct microbial community structures. MSS tumors consistently show enrichment of *F. nucleatum* and depletion of beneficial commensals such as Bacteroides species, whereas MSI-H tumors harbor greater overall microbial diversity and lower relative abundance of pathobionts (40, 53, 58). This differential microbial colonization directly shapes the predominant regulatory axes in each subtype: the Fap2–TIGIT and succinate–SUCNR1 inhibitory axes are more active in MSS, whereas the ZPS–LAG-3 and SCFA–TLR activating axes may be relatively preserved in MSI-H. From a therapeutic perspective, this divergence implies that pathogen-directed strategies—such as phage therapy targeting *F. nucleatum*—are more feasible and potentially more impactful in MSS, whereas MSI-H may benefit more from broad-spectrum microbiome enrichment approaches.

These four dimensions are not independent but functionally interconnected. The high abundance of *F. nucleatum* in MSS both drives succinate accumulation (metabolic dimension) and engages TIGIT binding (immune checkpoint dimension), while also physically hindering NK cell infiltration through recruitment of MDSCs and M2 macrophages, thereby contributing to spatial immune exclusion. Conversely, the greater microbial diversity in MSI-H may support a broader array of activating signals, contributing to the preservation of NK cell function despite the presence of inhibitory checkpoints. The convergence of multiple inhibitory mechanisms in MSS explains why monotherapeutic immunotherapeutic approaches are insufficient and why multimodal strategies—addressing microbial, metabolic, spatial, and immune checkpoint barriers sequentially—are theoretically justified.

Critically, these multidimensional biological distinctions translate directly into divergent clinical strategies. The low mutational burden in MSS explains why PD-1/PD-L1 blockade monotherapy fails; spatial immune exclusion explains why NK cell access to tumor cells is physically constrained and why spatial remodeling is more critical in MSS than in MSI-H; the succinate-rich metabolic environment explains why NK cells in MSS are actively suppressed rather than merely insufficient in number, justifying metabolite-targeted interventions; and the *F. nucleatum*-dominated microbial community provides a targetable upstream driver that is less prominent in MSI-H. Collectively, these features provide a clear rationale for the view that MSS tumors require a fundamentally different immunotherapeutic approach—one that targets the microbiota–NK axis through sequential remodeling of microbial, metabolic, spatial, and checkpoint barriers, rather than relying solely on neoantigen-driven T cell responses. **Table 6** summarizes this multidimensional comparison and its therapeutic implications.

**Table 6:** Multidimensional comparison of immune microenvironments in MSS versus MSI-H CRC and therapeutic implications.

Dimension	MSS	MSI-H	Therapeutic implication	Ref.
Mutational burden/neoantigens	Low	High	ICB ineffective in MSS; NK-targeted strategies needed as primary immune approach	(27, 110, 149)
Immune cell infiltration	Immune-desert/excluded	Immune-inflamed	Spatial reprogramming may yield greater benefit in MSS	(28, 116)
NK cell spatial distribution	Stroma-restricted, poor parenchymal penetration	Uniform distribution across stroma and parenchyma	“50 μm inhibitory zone” effect amplified in MSS; perivascular niche preservation more critical	(28, 116, 136, 150)
Microbial composition	*F. nucleatum* enriched, Bacteroides depleted	Higher diversity, lower *F. nucleatum*	Pathogen-directed interventions (phage, SUCNR1 antagonists) selectively feasible in MSS	(40, 53, 58)
Metabolic microenvironment	Succinate/lactate high, acidic pH	Metabolically permissive	Metabolite-targeted therapies (SUCNR1 blockade) specifically relevant in MSS	(61, 62, 67, 68)
Response to ICB	0–5%	30–50%	Microbiota-NK targeting fills therapeutic gap in MSS; ICB remains standard in MSI-H	(27, 149)

### 9.2 Why the microbiota–NK axis may be more central in MSS

The heightened relevance of the microbiota–NK axis in MSS arises from the convergence of three interrelated factors that extend beyond mutational burden alone.

#### 9.2.1 Neoantigen scarcity elevates the relative importance of innate immunity

In MSI-H tumors, neoantigen-driven T cell responses constitute the dominant force in antitumor immunity (27, 59); consequently, microbial modulation of NK cells may be overshadowed or diluted by this robust adaptive immune response (79). In MSS tumors, however, the scarcity of neoantigens significantly elevates the relative importance of innate immunity, particularly NK cells. In the absence of robust T cell attack, NK cells become one of the few mobilizable forces in antitumor defense. Therefore, targeting the microbiota to activate NK cells may yield greater marginal clinical benefit in MSS patients (26, 88).

#### 9.2.2 Spatial exclusion amplifies the value of spatial reprogramming

As noted, the physical restriction of immune cells to the tumor stroma is a hallmark feature of MSS that is far less prominent in MSI-H tumors (28, 116). The spatial patterns described in this framework—the “50-μm inhibitory zone” and “perivascular activation centers” (136, 150)—may be amplified in MSS. Consequently, spatial redistribution via strategies such as fecal microbiota transplantation to redirect beneficial bacteria to perivascular niches (144) may yield more pronounced efficacy gains in MSS patients (88). Moreover, the confinement of NK cells to the stroma in MSS implies that overcoming this physical barrier through microbial modulation of chemokine receptor expression (CXCR3, CCR5) and vascular permeability may translate directly into improved tumor accessibility and function.

#### 9.2.3 Microbial pathogenic drivers are more prominent in MSS

The consistent enrichment of *F. nucleatum* in MSS tumors (40, 58) establishes a direct mechanistic link between microbial dysbiosis and NK cell dysfunction through the Fap2–TIGIT and succinate–SUCNR1 axes. This dominance of pathogenic microbes provides a clearer therapeutic entry point—reducing *F. nucleatum* burden via phage therapy or blocking its downstream effectors may yield more substantial NK functional recovery in MSS than in MSI-H, where microbial drivers are more heterogeneous and less definitively pathogenic. Furthermore, the convergence of *F. nucleatum*-driven inhibition with metabolic (succinate) and spatial (MDSC recruitment) mechanisms creates a unified logic for intervention in MSS that is less applicable to the more heterogeneous MSI-H microenvironment.

### 9.3 Applicability boundaries of this framework in MSI-H

This framework is not without value for MSI-H patients, being primarily applicable in two contexts (1): patients who develop acquired resistance following ICI therapy, wherein *F. nucleatum* enrichment and NK cell dysfunction may emerge as novel immune evasion mechanisms (58); and (2) patients who are ineligible for or decline ICIs due to autoimmune disease risk, organ transplant status, or other contraindications. In these scenarios, the multidimensional MSS versus MSI-H comparison outlined above (**Table 6**) provides a reference for identifying when microbiota–NK modulation may become relevant in the MSI-H setting—particularly when acquired *F. nucleatum* colonization, metabolic shifts toward succinate accumulation, or spatial immune redistribution begin to recapitulate MSS-like features. Overall, however, ICIs remain the standard first-line therapy for MSI-H tumors; the role of this framework should therefore be positioned as “complementary” rather than “substitutive”.

### 9.4 Explicit research call: exclusive MSS-only cohorts

Given the pervasive patient heterogeneity in current microbiota–NK axis research, this review explicitly contends that the mixing of MSS and MSI-H patients in the vast majority of clinical studies (53, 59) is likely to dilute the true effects of microbiota–NK interactions and confound mechanistic attribution (151). To address this issue, prospective, multicenter collaborative studies enrolling exclusively MSS patients should be prioritized, eliminating the immune microenvironmental variability introduced by microsatellite instability status. The design of such studies should encompass at least five core dimensions to comprehensively capture the multidimensional differences outlined in Section 9.1:

(i) Baseline fecal metagenomic sequencing should cover bacteria, fungi, and archaea (53, 117) to comprehensively capture cross-kingdom microbial features, with particular attention to the abundance of *F. nucleatum* and *B. uniformis* as prototypical pathogenic and beneficial drivers, respectively.

(ii) Spatial transcriptomics or multiplex immunohistochemistry of tumor tissues should be performed to enable simultaneous visualization of NK cell subset markers (GZMB, PD-1, TIGIT, LAG-3, CD45RO, IL-10) and spatial localization of key pathogens (*F. nucleatum*, *B. uniformis*), and to quantify the “50-μm inhibitory zone” and perivascular activation center phenomena (24, 136).

(iii) Host genetic analysis should be incorporated, focusing on polymorphisms in immune-related genes such as TIGIT (rs7920644), LAG-3 (rs870849), and SUCNR1 (rs13079765), which may modulate pathway efficiency and stratify patients for pathway allocation (12, 63, 109).

(iv) Metabolomic profiling of tumor tissues and/or fecal samples should be added to quantify succinate, lactate, and short-chain fatty acid levels, enabling direct assessment of the metabolic microenvironment’s contribution to NK cell dysfunction and providing baseline data for metabolite-targeted interventions (62, 67).

(v) Clinical outcomes should be documented using standardized criteria, with unified assessment of progression-free survival, overall survival, and therapeutic responses to ICIs or chemotherapy, to enable comparable integrated analysis of multimodal data within a unified framework (59, 149).

These five dimensions collectively contribute to a comprehensive systems-level understanding of the microbiota–NK axis in MSS, transcending mutation-centric unidimensional comparisons to capture the spatial, metabolic, microbial, and genetic heterogeneity that determines patient outcomes and therapeutic responses.

### 9.5 Repositioning of the two precision pathways

Based on the foregoing analysis, the primary target population for the “enrichment” and “remodeling” strategies proposed in Section 8 should be MSS patients. The multidimensional comparative framework (**Table 6**) provides the rationale for this repositioning: MSS tumors combine low mutational burden (rendering T cell-directed therapies less effective), spatial immune exclusion (making spatial reprogramming more critical), *F. nucleatum*-driven microbial pathogenesis (providing clear intervention targets), and an unfavorable metabolic microenvironment (amplifying NK dysfunction). These four features collectively establish MSS as the subtype in which microbiota–NK axis modulation is most likely to translate into clinical benefit.

The remodeling strategy should be positioned as a first-line immune intervention option for MSS patients rather than a second-line alternative (88, 143). For MSS patients with high *F. nucleatum* abundance and predominant TIGIT^+^ dysfunctional NK cells (56), a sequential regimen—commencing with phage therapy, followed by SUCNR1 antagonism, and concluding with TIGIT blockade (75, 139, 143)—may represent a vital approach to addressing the therapeutic vacuum following ICI failure (27). In this context, the metabolic dimension becomes actionable: reducing succinate through SUCNR1 antagonism not only reverses NK dysfunction but also alleviates the acidic microenvironment that impairs other immune effectors, creating a permissive environment for subsequent TIGIT blockade. The spatial dimension is also addressed: phage-mediated reduction of *F. nucleatum* may alleviate MDSC/M2 macrophage recruitment, thereby mitigating physical barriers to NK cell infiltration.

The enrichment strategy is applicable to the MSS subset with pre-existing favorable microbiota–NK features, including high abundance of *B. uniformis* and predominant activated NK cells (26), with the goal of maintaining an immune “hot” state in these patients (88). For these patients, sustaining the perivascular activation niche through SCFA supplementation or ZPS-based interventions may maintain NK cell access to tumor sites and prevent the emergence of spatial exclusion barriers. Here, the metabolic dimension plays a supportive role: SCFAs not only directly activate NK cells but also maintain intestinal barrier integrity, limiting the translocation of pathobionts that could disrupt favorable microbial ecosystems.

Critically, selection between the two pathways should be guided by baseline multidimensional profiling (**Table 7**). Patients with high *F. nucleatum*, elevated succinate, pronounced stromal immune exclusion, and predominant TIGIT^+^ NK cells are candidates for the remodeling strategy. Conversely, patients with high *B. uniformis*, preserved perivascular NK aggregation, low succinate, and predominant LAG-3^+^ activated NK cells are candidates for the enrichment strategy. This multidimensional allocation framework ensures that pathway selection is based on the full complexity of the MSS microenvironment rather than any single biomarker, directly responding to the multidimensional nature of MSS versus MSI-H differences detailed in Section 9.1.

**Table 7:** Multidimensional profiling for pathway allocation in MSS patients.

Profiling dimension	Enrichment strategy candidate	Remodeling strategy candidate
Microbial	High *B. uniformis*, low *F. nucleatum*	High *F. nucleatum*, low *B. uniformis*
Metabolic	Low succinate/lactate, high SCFAs	High succinate/lactate, low SCFAs
NK spatial	Perivascular clustering, LAG-3^+^ dominant	Stromal exclusion, TIGIT^+^ dominant
NK functional	GZMB^+^IFN-γ^+^ activated	PD-1^+^TIGIT^+^ dysfunctional
Genetic	LAG-3 rs870849 GG favorable	TIGIT rs7920644 or SUCNR1 rs13079765 risk alleles
Intervention logic	Enhance pre-existing immunity	Relieve suppression then activate

### 9.6 Summary of the MSS multidimensional framework

In summary, the revised MSS versus MSI-H comparison presented in this section transcends the mutation-centric reductionist view, embracing a multidimensional framework encompassing mutational burden, spatial immune architecture, metabolic microenvironment, and microbial colonization profiles. This expanded perspective: (i) explains why the microbiota–NK axis is disproportionately relevant in MSS through the convergence of three distinct mechanistic rationales; (ii) provides a theoretical foundation for spatially and metabolically co-targeted strategies alongside microbial and checkpoint modulation; (iii) establishes specific, measurable dimensions for patient stratification (**Table 7**); and (iv) outlines specific analytical requirements for future MSS-only prospective cohorts. Critically, these multidimensional biological distinctions directly dictate divergent therapeutic strategies—MSS requires a multimodal sequential approach targeting microbial, metabolic, spatial, and immune barriers, whereas MSI-H is primarily driven by neoantigen-directed T cell responses that are effectively addressed by ICI monotherapy. We contend that this multidimensional framework provides a more robust and clinically actionable foundation for translating insights from the microbiota–NK axis into therapeutic benefits for MSS patients than previous unidimensional approaches.

## 10 Unresolved questions and future directions

### 10.1 Five key unresolved questions

#### 10.1.1 Causality versus correlation

Spatial transcriptomics reveals NK cell dysfunction in proximity to *F. nucleatum*, yet whether *F. nucleatum* directly causes dysfunction or whether dysfunctional NK cells permit *F. nucleatum* overgrowth remains unresolved. No study to date has employed time‑resolved sampling or experimental perturbation, such as clearing *F. nucleatum* in humans and observing subsequent NK functional changes, to definitively establish causal direction (143). Organoid coculture systems combined with live‑cell imaging and single‑cell resolution may help address this question (152, 153).

#### 10.1.2 Reversibility

Can established dysfunctional NK cells revert to a functional state, or are they terminally differentiated? Mouse models suggest partial reversibility with TIGIT blockade (56), but human data remain lacking. Single‑cell Assay for transposase accessible chromatin using sequencing (ATAC‑seq) and lineage‑tracing studies could provide valuable insights into this issue (15, 154).

#### 10.1.3 The LAG-3 paradox

Why does LAG-3 activation enhance NK cell function yet inhibit T cells? The answer may lie in differential adaptor usage, with FcϵRIγ on NK cells versus SHP‑1/SHP‑2 on T cells, requiring direct biochemical validation through CRISPR‑based modification of primary human lymphocytes (26).

#### 10.1.4 MSS‑specific causal validation

Prospective cohorts exclusively enrolling MSS patients (59), combined with time‑resolved sampling and experimental perturbation (143), are needed to directly validate the causal role of the microbiota–NK axis in MSS (151).

#### 10.1.5 Cross‑kingdom biomarkers

Can the addition of fungal and archaeal markers to bacterial panels improve prediction of immunotherapy responses over current metrics? This requires prospective cohorts with metagenomic sequencing covering all three kingdoms, coupled with NK subset phenotyping and clinical outcome tracking (117, 121).

### 10.2 Methodological breakthrough: AI‑driven multi‑omics integration

The field has now accumulated massive, high-dimensional, heterogeneous, and multi-scale datasets, encompassing microbiome, transcriptomic, spatial, metabolomic, and host genetic profiles. Traditional statistical methods are often inadequate for capturing the nonlinear associations and interaction information inherent in such complex data, whereas machine learning approaches, particularly random forest, support vector machines, and deep learning models, are increasingly being applied to the systematic integration of multi-omic profiles (155, 156).

In specific application scenarios, when combined with time-series sampling and structural causal models, artificial intelligence can be used to infer causal directions between microbial dynamics and NK cell functional changes, moving beyond simple correlation analysis. For MSS-specific CRC, constructing prediction models exclusively for these patients allows direct testing of the independent prognostic power of microbiota–NK axis biomarkers. In cross-kingdom biomarker discovery, deep learning models can automatically learn higher-order interaction features among bacteria, fungi, and archaea, identifying combinatorial biomarkers that transcend single kingdoms (155, 156).

However, it must be clearly stated that the above AI models are currently in the exploratory research phase, and their clinical translation requires rigorous prospective cohort validation, a priority for the next phase of this field.

### 10.3 Preventive potential

The microbiota–NK axis also holds significant, yet underappreciated, preventive potential (157). Microbiota‑based interventions can be safely administered to high‑risk populations over extended periods (158, 159). Functional foods containing *B. uniformis* (26), SCFA precursors such as resistant starch and inulin (80, 81), nicotinamide (90), or combinations thereof, could be tested in individuals at elevated CRC risk, including those with familial adenomatous polyposis (40), Lynch syndrome (110), long‑standing inflammatory bowel disease (160), or prior adenoma resection (121). The goal is to maintain a landscape dominated by activated and memory‑like NK subsets while preventing the emergence of *F. nucleatum*‑driven dysfunctional cell populations (12, 58).

## 11 Conclusion

The microbiota–NK cell axis in CRC has evolved from a descriptive observation into a mechanistically defined, spatially organized, and potentially actionable system (161–163). By shifting the framework from a linear, pathogen-centric view to a subset-centric and spatially resolved perspective, this review integrates multiple advances from the existing literature (162, 164). It must be candidly stated, however, that these advances still largely rest on hypothesis-driven framework integration rather than a mature body of knowledge (162, 165). The spatial transcriptomics-derived concepts of “inhibitory zone” and “activation center” offer new perspectives on microbe–NK interactions, but the current evidence base remains limited to small-scale exploratory studies and is insufficient to support clinical decision-making (141). The core value of this framework lies in integrating fragmented evidence and generating testable hypotheses, rather than providing ready-to-use clinical protocols (140).

The main contributions of this review are: (i) replacing the general concept of NK cell infiltration with functional subset classification, each subset driven by distinct microbial signals and carrying different prognostic and therapeutic implications (13, 37, 166); (ii) introducing spatial resolution as a key determinant, showing that microbe–NK proximity, rather than mere abundance, determines functional outcomes (165, 167); (iii) recognizing metastatic site differences in pathogen–NK associations (166, 168); (iv) translating mechanistic and spatial insights into two clinically testable precision pathways, namely enrichment and remodeling strategies (6, 135); and (v) systematically discussing key uncertainties and translational barriers within the current framework, including unresolved causality, the LAG-3 paradox, small spatial evidence samples, unclear microbial drivers of regulatory NK cells, and the unresolved initial differentiation mechanisms of memory-like NK cells (162).

Acknowledging these limitations, the microbiota–NK axis offers a perspective worthy of further exploration for improving outcomes in immunotherapy-resistant CRC, particularly the MSS subtype (169). The shift from description to mechanism, from bulk to spatial, and from generic to hypothesis-driven frameworks opens new possibilities for future research in this challenging patient population (162, 164). The value of this framework lies in guiding subsequent clinical trial designs, not in replacing current standard of care (140).
